# Sex differences in psychiatric diagnoses preceding autism diagnosis and their stability post autism diagnosis

**DOI:** 10.1111/jcpp.14130

**Published:** 2025-02-28

**Authors:** Miriam I. Martini, Ralf Kuja‐Halkola, Agnieszka Butwicka, Ebba Du Rietz, Aleksandra Kanina, Isabell Brikell, Zheng Chang, Henrik Larsson, Paul Lichtenstein, Sven Bölte, Francesca Happé, Mark J. Taylor

**Affiliations:** ^1^ Department of Medical Epidemiology and Biostatistics Karolinska Institutet Stockholm Sweden; ^2^ Division of Mental Health Services, R&D Department Akershus University Hospital Lørenskog Norway; ^3^ Institute of Clinical Medicine, Faculty of Medicine University of Oslo Oslo Norway; ^4^ Department of Biostatistics and Translational Medicine Medical University of Lodz Lodz Poland; ^5^ Department of Global Public Health and Primary Care University of Bergen Bergen Norway; ^6^ Department of Biomedicine Aarhus University Aarhus Denmark; ^7^ School of Medical Sciences Örebro University Örebro Sweden; ^8^ Department of Women's and Children's Health, Center of Neurodevelopmental Disorders (KIND), Centre for Psychiatry Research Karolinska Institutet & Stockholm Health Care Services, Region Stockholm Stockholm Sweden; ^9^ Child and Adolescent Psychiatry Stockholm Health Care Services, Region Stockholm Stockholm Sweden; ^10^ Curtin Autism Research Group, Curtin School of Allied Health Curtin University Perth WA Australia; ^11^ Social, Genetic and Developmental Psychiatry Centre, Institute of Psychiatry, Psychology, and Neuroscience King's College London London UK

**Keywords:** Autism spectrum disorder, sex differences, psychiatric diagnoses, follow‐up study

## Abstract

**Background:**

Autistic individuals often receive psychiatric diagnoses prior to their autism diagnosis. It remains unclear to what extent autistic females and males differ in their likelihood of receiving psychiatric diagnoses prior to their autism diagnosis and continue seeking care for them after an autism diagnosis.

**Methods:**

In a nationwide cohort of all individuals born in Sweden 1990–2015 with a clinical autism diagnosis (*N* = 72,331, *n*_females_ = 24,110), we used linear and logistic regression to estimate the association between sex and (a) psychiatric diagnoses before autism diagnosis, including time trends by autism diagnosis year (2010–2020), (b) autism diagnosis age in those with preceding diagnoses, (c) stability of preceding diagnoses (defined as continued care utilization indicated through diagnosis or medication in the 5 years following autism diagnosis).

**Results:**

In total 54.2% of autistic females and 40.9% of autistic males received at least one preceding psychiatric diagnosis (most common: ADHD, anxiety, depression). Autistic females showed higher odds than males for most preceding psychiatric diagnoses (OR_range_ = 1.29 [1.18, 1.41]–10.69 [8.06, 14.17]), except psychotic disorders (OR = 0.91 [0.78, 1.06]) and ADHD (OR = 0.69 [0.66, 0.71]). Sex differences in preceding diagnoses were persistent across different autism diagnosis years (2010–2020). For most conditions, females with a preceding diagnosis were diagnosed with autism later than males with the same condition. For both sexes, the stability of preceding diagnoses varied considerably (23.1%–88.9%) and was less than 50% for most diagnoses. Females showed a higher stability for anxiety, sleep disorders and self‐harm (OR_range_ = 1.45 [1.30, 1.62]–2.37 [1.93, 2.90]), and males for psychotic disorders (OR = 0.60 [0.44, 0.81]).

**Conclusions:**

Autistic females are more likely to be diagnosed with psychiatric conditions prior to an autism diagnosis and receive care for them post autism diagnosis. Our findings emphasize the variability of clinical presentation and importance of disentangling persistent support needs from overlapping diagnostic presentations, particularly in autistic females, to provide appropriate and timely care.

## Introduction

Autistic individuals face significant challenges regarding their mental health (Lai et al., 2019). They are more likely to be diagnosed with and hospitalized for psychiatric conditions than nonautistic individuals (Martini et al., 2022). This is particularly true for autistic females (Lai et al., 2019; Martini et al., 2022; Rødgaard et al., 2021a). Many autistic individuals are diagnosed with psychiatric disorders in early childhood (Rødgaard et al., 2021a; Simonoff et al., 2008) and often prior to being diagnosed with autism (Aggarwal & Angus, 2015; Fusar‐Poli, Brondino, Politi, & Aguglia, 2022; Geurts & Jansen, 2012; Kentrou, Livingston, Grove, Hoekstra, & Begeer, 2024; Kentrou, Oostervink, Scheeren, & Begeer, 2021). Autism may remain undetected until referral to mental health services for psychiatric difficulties (Aggarwal & Angus, 2015; Lai & Baron‐Cohen, 2015). These findings underscore the complexity of diagnosing autism in the context of co‐occurring mental health issues and emphasize the need for improved early identification.

Similarly, individuals referred for autism assessment show previous contact with mental health services (Aggarwal & Angus, 2015; Fusar‐Poli et al., 2022; Geurts & Jansen, 2012), with eating disorders as one example where autistic women frequently receive treatment for a mental health problem before autism is recognized (Babb et al., 2022). In studies from the Netherlands (*N* = 1,019–1,211), 43% to 50% of autistic individuals self‐reported having received a psychiatric diagnosis prior to their autism diagnosis (Kentrou et al., 2021, 2024). While these findings provide valuable insights, registered diagnoses may offer a more objective and potentially more reliable source of diagnostic information. Preceding diagnoses appear more common in autistic females than males (Geurts & Jansen, 2012; Gu, Dawson, & Engelhard, 2023; Kentrou et al., 2021). However, existing studies (Geurts & Jansen, 2012; Kentrou et al., 2024, 2021; Rødgaard et al., 2021b) have predominantly been based on smaller samples of late‐diagnosed adults, warranting a population‐based investigation of sex differences across a comprehensive list of preceding psychiatric diagnoses including younger autistic individuals. Additionally, with increased numbers of females diagnosed in recent years (Jensen, Steinhausen, & Lauritsen, 2014; Russell et al., 2022), examining changes in sex differences of preceding diagnoses over time is warranted.

Few studies have directly examined sex differences in the timing of autism diagnosis in those with and without preceding psychiatric diagnoses. Although females are increasingly diagnosed with autism (Jensen et al., 2014; Solmi et al., 2022), they still tend to receive their diagnosis later than autistic males (Begeer et al., 2013; Giarelli et al., 2010; McDonnell et al., 2021). Diagnoses of psychiatric conditions might delay autism diagnosis, particularly in females (Kentrou, de Veld, Mataw, & Begeer, 2019; Petrou, Parr, & McConachie, 2018). A study using health records from 1,438 autistic individuals diagnosed between 2014 and 2021 showed that adjusting for co‐occurring conditions such as anxiety and mood disorders, which were more frequent in autistic females, attenuated the association between sex and age at autism diagnosis (Gu et al., 2023). Nevertheless, contact with psychiatric care for non‐autism‐specific challenges might facilitate identifying autism in females (Duvekot et al., 2017; Dworzynski, Ronald, Bolton, & Happé, 2012), highlighting the need to further examine the complex relationship between psychiatric diagnoses and autism recognition alongside a careful consideration of sex differences in clinical pathways.

Diagnosis of psychiatric conditions in autistic individuals is complicated by the overlap of autistic traits with symptoms and diagnostic criteria of psychiatric conditions, for example, anxiety (e.g. anxiousness in social situations), eating disorders (e.g. inflexibility in cognitions and routines) or attention‐deficit hyperactivity disorder (ADHD, e.g. regulation of attention) (Brede et al., 2020; Fusar‐Poli et al., 2022; Happé et al., 2016; Lai & Baron‐Cohen, 2015). Autistic traits might be understood as symptoms of these conditions leading to a diagnosis that later turns out to be suboptimal (misdiagnosis) or, if both conditions are present, attributed to a psychiatric diagnosis (diagnostic overshadowing) (Fusar‐Poli et al., 2022). In a small sample (*N* = 161), almost half of the autistic individuals retained their preceding diagnosis (Fusar‐Poli et al., 2022), and co‐occurring psychiatric diagnoses are common (Kentrou et al., 2021; Martini et al., 2022). At the same time, studies from the Netherlands found that 40% of autistic individuals (*N* = 1,019; Kentrou et al., 2021) self‐reported a preceding diagnosis that was no longer present following autism diagnosis, and almost 25% (*N* = 1,211) perceived their preceding psychiatric diagnosis to be a misdiagnosis (Kentrou et al., 2024). These percentages were higher for autistic females (47.0% and 31.7%) than males (27.3% and 16.7%). Importantly, this does not preclude other persistent psychiatric difficulties over time. Understanding the persistence of preceding psychiatric diagnoses in the few years following an autism diagnosis can provide insights into the validity of these prior diagnoses; whether they represent ongoing, continuous – rather than new – episodes of psychiatric conditions that require sustained care or whether they reflect diagnostic overshadowing or a misdiagnosis that becomes clarified once autism is diagnosed.

The current study therefore aimed to investigate sex differences in (a) the extent to which autistic females and males receive psychiatric diagnoses preceding an autism diagnosis, including time trends between 2010 and 2020, (b) how preceding diagnoses are associated with age at autism diagnosis, and (c) the extent to which prior diagnoses are stable (requiring continued care in specialist psychiatry) in the 5 years following an autism diagnosis.

## Methods

### Study population

For this longitudinal, population‐based cohort study we linked data from Swedish national registers with follow‐up until 31st December 2020. Legally registered sex (male/female) was obtained from the Total Population Register. Using information from the Medical Birth Register (MBR; Axelsson, 2003) and the National Patient Register (NPR; Ludvigsson et al., 2011), which covers specialist psychiatric inpatient care from 1973 and outpatient care from 2001, we identified all individuals born in Sweden between 1990 and 2015 with an autism diagnosis (*N* = 75,702). We excluded individuals with congenital chromosomal syndromes, individuals who died or emigrated before the end of the study, and whose biological mother could not be identified in the registers (eligible cohort of 72,331, Figure S1).

We followed the Strengthening the Reporting of Observational Studies in Epidemiology (STROBE) reporting guideline. The study was approved by the Swedish Ethical Review Authority (Dnr 2020–06540). Informed consent is not required for register studies in Sweden.

### Measures

Autism diagnosis was defined as receiving at least one diagnosis of ICD‐9 code 299A (Autism), ICD‐10 codes F84.0 (Childhood Autism), F84.1 (Atypical Autism), F84.5 (Asperger syndrome), F84.8 (Other pervasive developmental disorders), or F84.9 (Pervasive developmental disorder not otherwise specified) in the NPR. Age at autism diagnosis was calculated based on the first recorded autism diagnosis date after age 1. In Sweden, autism diagnoses are typically assigned within specialized child and adolescent psychiatric services or adult psychiatric care. The diagnostic process is comprehensive, involving detailed developmental history, psychological assessments and a medical evaluation by a trained (child) psychiatrist in predominantly outpatient settings.

We extracted outpatient and inpatient diagnoses of 10 psychiatric disorders, including self‐harm and ADHD using the discharge date for the first recorded diagnosis for each condition. For readability, we grouped ADHD with other psychiatric diagnoses, despite being a neurodevelopmental rather than a psychiatric condition. Using dispensed medication prescriptions with disorder‐specific indications in the Prescribed Drug Register (PDR; Furu et al., 2010; Wettermark et al., 2007), we identified additional individuals with ADHD, psychotic, bipolar, and sleep disorders. Diagnoses of intellectual disability (ID) were identified from the NPR. ICD and ATC codes are listed in Table S1.

Preceding diagnoses were defined as psychiatric diagnoses whose first recorded occurrence (by diagnosis or medication prescription) predated the first recorded autism diagnosis. Stability of preceding psychiatric diagnoses among autistic individuals with an existing prior diagnosis was defined as being seen in specialist care for the same psychiatric condition (indicated by registered diagnosis or relevant medication prescription) at least once within a maximum of 5 years after the initial recorded autism diagnosis.

### Statistical analyses

Data management was performed in SAS (version 9.4.6). Data were analyzed using R (version 4.2.3) with the drgee (Zetterqvist & Sjölander, 2015) package between October 2022 and July 2024. Regression analyses were implemented as generalized estimating equations (GEE) to account for related individuals in the dataset. We compared preceding psychiatric diagnoses between autistic females and males (reference group) using a logistic regression model. Associations are presented as odds ratios (ORs) for any preceding psychiatric diagnosis and each psychiatric diagnosis separately. We fitted a crude model and a model adjusted for birth year and age at first recorded autism diagnosis using natural cubic splines with 5 degrees of freedom. We performed separate analyses for autistic individuals with ID. Using logistic regression, we examined time trends in preceding diagnoses through the association between preceding diagnoses and the interaction between sex and year of autism diagnosis (2010–2020, natural cubic splines with 5 degrees of freedom) with and without adjustment for age at autism diagnosis. The birth‐year‐adjusted association between preceding diagnoses and the mean age at first recorded autism diagnosis was investigated using linear regressions. Among females and males with a preceding diagnosis, for each psychiatric diagnosis, we further calculated ORs comparing the odds of retaining the diagnosis within 5 years following an autism diagnosis. ORs were adjusted for birth year and autistic individuals with ID were analyzed separately.

We adjusted for multiple testing using the Benjamini‐Yekuteli False Discovery Rate (FDR) adjustment, resulting in a threshold of *p* = .004 for the main analysis. A cluster robust sandwich estimator was used to calculate confidence intervals (CIs) which account for related individuals in the sample. We performed several sensitivity analyses described in more detail in Table S2.

## Results

### Cohort description

We identified 72,331 autistic individuals, including 24,110 females (33.3%) and 48,221 males (Table 1) aged 5–30 years at the end of follow‐up. Information on race and ethnicity was not available. The mean age at first recorded autism diagnosis (*M* = 13.0 years, *SD* = 6.00, range = 1.0–30.9) was higher in females (14.6 years) than males (12.2 years). Table S3 shows the mean age by diagnosis year and sex. A total of 10,331 autistic individuals (14.3%, 29.6% females) were diagnosed with ID. For those with ID, the mean age of diagnosis was 10.9 years for females and 9.3 years for males.

**Table 1:** Descriptive characteristics of the study cohort

	Overall	Autistic males	Autistic females	*p*
*N* (%)	72,331	48,221 (66.7)	24,110 (33.3)	
Birth year (%)				
1990–1999	29,357 (40.6)	18,512 (38.4)	10,845 (45.0)	
2000–2009	33,354 (46.1)	22,225 (46.1)	11,129 (46.2)	
2010–2015	9,620 (13.3)	7,484 (15.5)	2,136 (8.9)	
Intellectual disability				
Diagnosis (%)	10,331 (14.3)	7,277 (15.1)	3,054 (12.7)	
Age at autism diagnosis				
Mean (*SD*)	13.01 (6.00)	12.22 (5.93)	14.60 (5.85)	<.001
Preceding psychiatric diagnosis: *N* (%)				
Any psychiatric disorder	32,774 (45.3)	19,700 (40.9)	13,074 (54.2)	<.001
ADHD	19,133 (26.5)	13,226 (27.4)	5,907 (24.5)	<.001
Anxiety disorders	11,181 (15.5)	4,669 (9.7)	6,512 (27.0)	<.001
Depressive disorders	9,671 (13.4)	4,511 (9.4)	5,160 (21.4)	<.001
Obsessive‐compulsive disorder	2,143 (3.0)	1,130 (2.3)	1,013 (4.2)	<.001
Bipolar disorder	915 (1.3)	379 (0.8)	536 (2.2)	<.001
Psychotic disorders	754 (1.0)	447 (0.9)	307 (1.3)	<.001
Anorexia nervosa	570 (0.8)	73 (0.2)	497 (2.1)	<.001
Other eating disorders	1,150 (1.6)	198 (0.4)	952 (3.9)	<.001
Sleep disorders	6,409 (8.9)	3,403 (7.1)	3,006 (12.5)	<.001
Self‐harm	2,756 (3.8)	1,078 (2.2)	1,678 (7.0)	<.001
Borderline personality disorder	474 (0.7)	56 (0.1)	418 (1.7)	<.001
Stable psychiatric diagnosis among those with a preceding diagnosis: *N* (%)				
ADHD	17,000 (88.9)	11,749 (88.8)	5,251 (88.9)	.920
Anxiety disorders	5,708 (51.1)	2,086 (44.7)	3,622 (55.6)	<.001
Depressive disorders	4,724 (48.8)	2,155 (47.8)	2,569 (49.8)	.050
Obsessive‐compulsive disorder	1,138 (53.1)	605 (53.5)	533 (52.6)	.700
Bipolar disorder	499 (54.5)	205 (54.1)	294 (54.9)	.873
Psychotic disorders	342 (45.4)	225 (50.3)	117 (38.1)	.001
Anorexia nervosa	222 (38.9)	19 (26.0)	203 (40.8)	.022
Other eating disorders	348 (30.3)	46 (23.2)	302 (31.7)	.023
Sleep disorders	2,246 (35.0)	1,031 (30.3)	1,215 (40.4)	<.001
Self‐harm	636 (23.1)	148 (13.7)	488 (29.1)	<.001
Borderline personality disorder	248 (52.3)	19 (33.9)	229 (54.8)	.005

ADHD, attention‐deficit hyperactivity disorder; *SD*, standard deviation; Stable psychiatric diagnoses are defined as receiving specialist psychiatric care or a prescription for medication for the same mental health problem within 5 years following an autism diagnosis.

### Preceding diagnoses

A total of 54.2% of autistic females and 40.9% of autistic males received at least one preceding psychiatric diagnosis, with ADHD, anxiety, and depressive disorders being the most common (Table 1, Figure S2). Among autistic females 28.3% received two or more preceding diagnoses of different conditions compared to 13.0% of males (Table S4).

**Figure 1:**
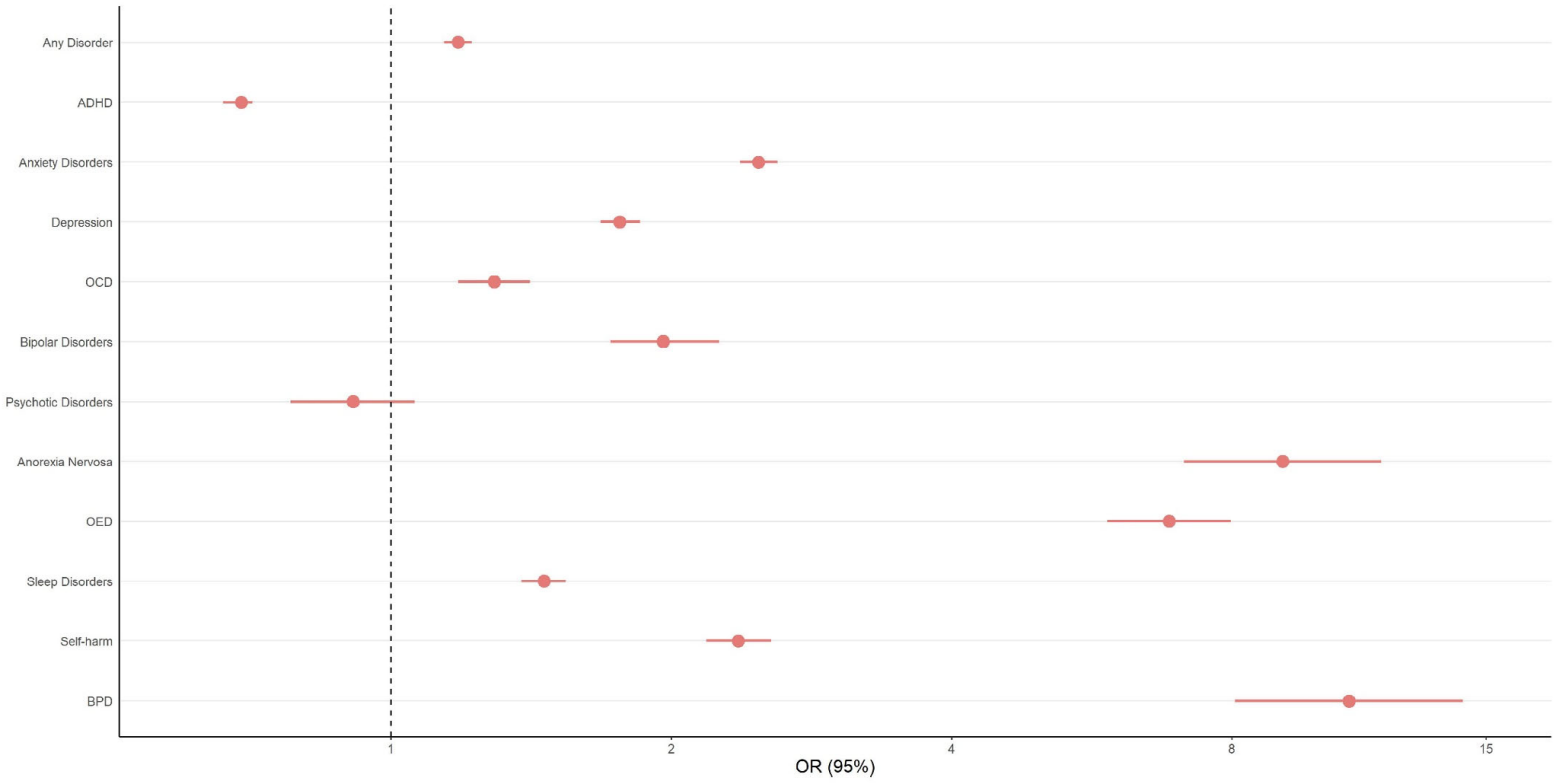
OR of preceding psychiatric diagnoses comparing autistic females and males. Males are the reference group. ADHD, attention‐deficit hyperactivity disorder; BPD, borderline personality disorder; OCD, obsessive‐compulsive disorder; OED, other eating disorders

Compared to males, females had statistically significantly increased odds for any (OR_adjusted_ = 1.18 [95% CI, 1.14, 1.22]) and most individual preceding psychiatric diagnoses (OR_range_ = 1.29 [1.18, 1.41]–10.69 [8.06, 14.17]) except psychotic disorders (OR_adjusted_ = 0.91 [0.78, 1.06]), and statistically significantly lower odds for ADHD (OR_adjusted_ = 0.69 [0.66, 0.71]; Figure 1, Table S5). Sensitivity analyses in those with full 5‐year coverage prior to autism diagnosis and those diagnosed with autism before age 18 showed the same pattern. When only including individuals born after 2000, the association of sex with bipolar disorder was non‐significant after multiple testing adjustment (Table S6). Considering sex differences in groups diagnosed with autism at different ages, we observe increased odds for females compared to males across most diagnoses in those diagnosed with autism between 9–15, 16–24 and 25 and older but fewer differences in those diagnosed with autism between ages 1 and 8 (Table S7).

Among autistic individuals with ID, we observed lower prevalences of prior diagnoses compared to autistic individuals without ID (any preceding diagnosis: 29.6% females, 25.2% males; Table S8). Autistic females with ID had higher odds for most preceding psychiatric diagnoses except for OCD, psychotic and sleep disorders than autistic males with ID (Table S9).

### Preceding diagnoses over time

**Figure 2:**
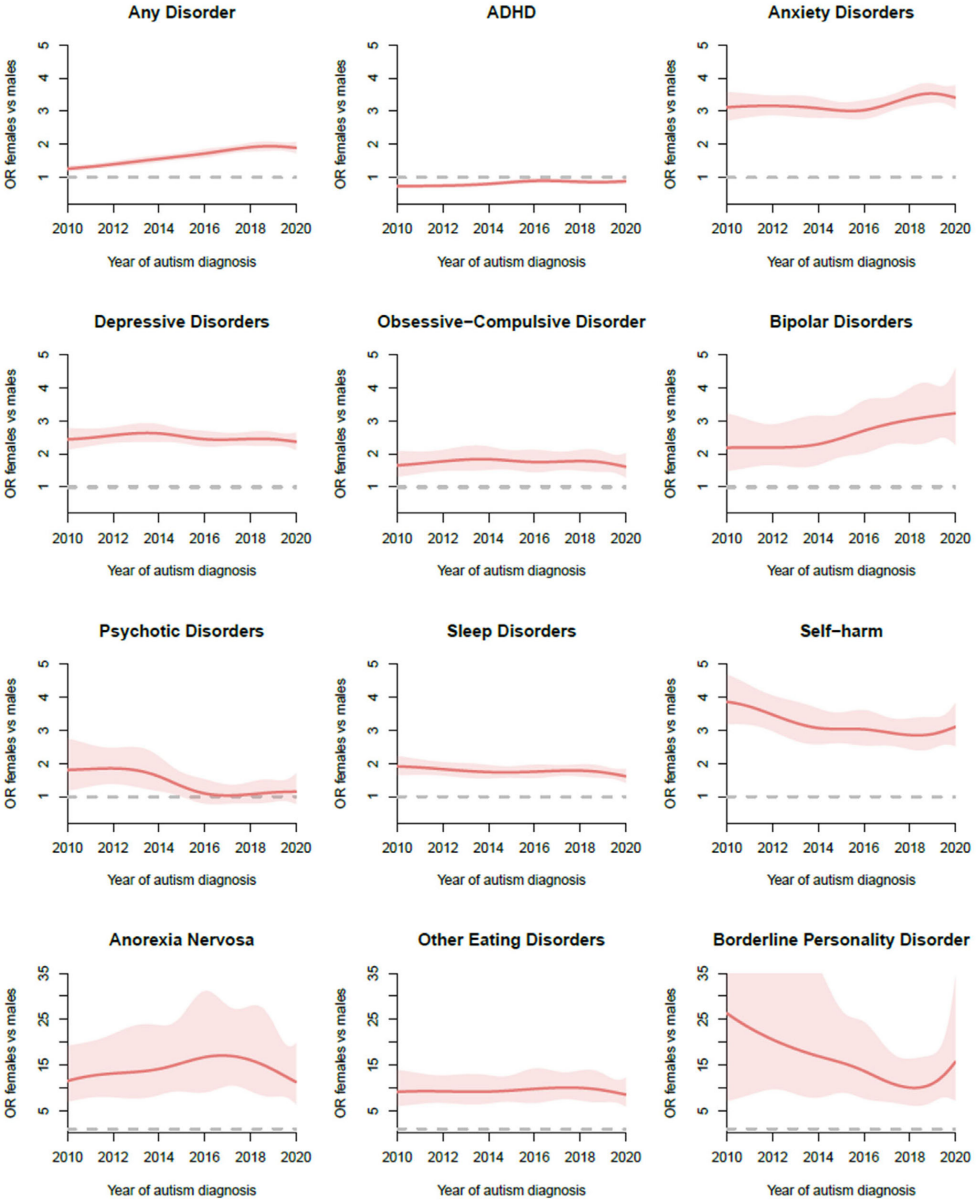
OR comparing preceding diagnoses between females and males diagnosed with autism between 2010 and 2020. Males are the reference group. Please note the difference in *y* axis for anorexia nervosa, other eating disorders and borderline personality disorder. ADHD, attention‐deficit hyperactivity disorder

The probability of receiving a preceding psychiatric diagnosis across autism diagnosis years 2010–2020 by sex is shown in Figure S3. Most diagnoses increased in probability over time, except for ADHD, psychotic disorders and bipolar disorders in males. We observed consistent sex differences over time, with females diagnosed with autism from 2010 to 2020 showing higher odds than males for preceding diagnoses of most conditions except ADHD and psychotic disorders (Figure 2), even when adjusting for autism diagnosis age (Figure S4).

### Association with the timing of autism diagnosis

**Figure 3:**
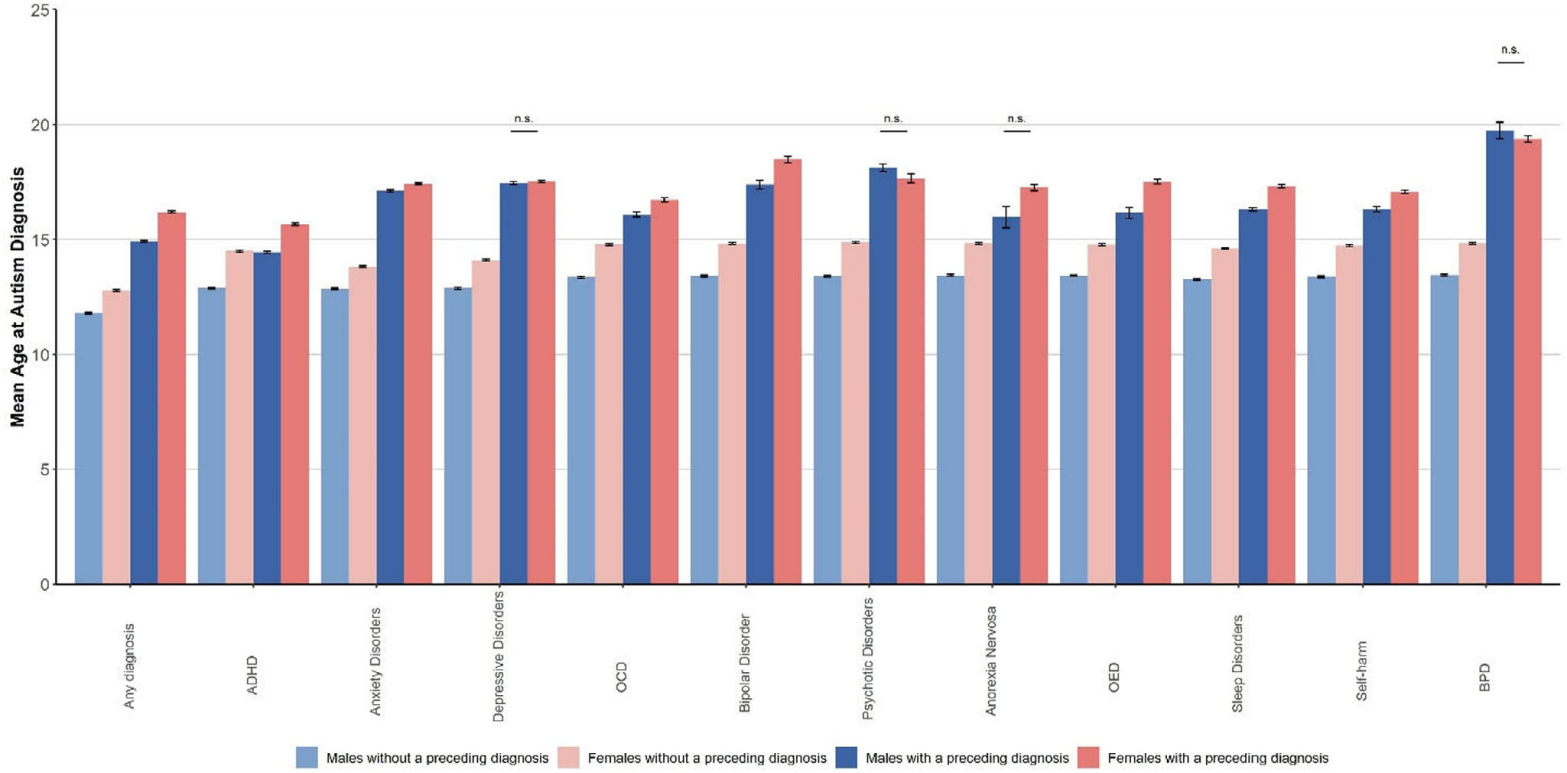
Mean age and standard errors at autism diagnosis adjusted for birth year by sex and preceding diagnosis. This figure shows the birth‐year‐adjusted mean age at autism diagnosis, including standard errors. Group comparisons within and between sexes are statistically significant unless otherwise indicated. ADHD, attention‐deficit hyperactivity disorder; BPD, borderline personality disorder; OCD, obsessive‐compulsive disorder; OED, other eating disorders

The mean age of autism diagnosis by sex and preceding diagnosis, with and without adjustment for birth year, are shown in Table S10. Among those without preceding diagnoses, females were consistently diagnosed later than males (range = 0.96–1.61 years). Females with any preceding diagnosis were diagnosed at a statistically significantly higher age than males (difference_adjusted_ = 1.27 years). This pattern was also observed for most individual preceding psychiatric diagnoses except for psychotic disorders, anorexia nervosa, depression and borderline personality disorder (BPD), for which we observed no difference in mean age between females and males (Figure 3, Table S11). The pattern was largely unchanged in individuals diagnosed before age 18 and those born after 2000 (Table S12), except for a statistically significant sex difference for depression. In individuals with ID, females with any preceding diagnoses and preceding diagnoses of ADHD, bipolar disorder, other eating disorders, sleep disorder and self‐harm were consistently diagnosed later than males with these diagnoses (Table S13).

### Stability of preceding diagnoses

Depending on the condition, 23.1% (self‐harm) to 88.9% (ADHD) of individuals with a preceding diagnosis were seen by a specialist for the same condition within 5 years after autism diagnosis (registered diagnosis/prescribed medication), indicating variability across different conditions. Only 5 out of 11 conditions reached stability above 50% (ADHD, anxiety, OCD, bipolar disorders, BPD). ADHD was the most stable diagnosis in both autistic females (88.8%) and males (88.9%; Table 1, Figure S2).

**Figure 4:**
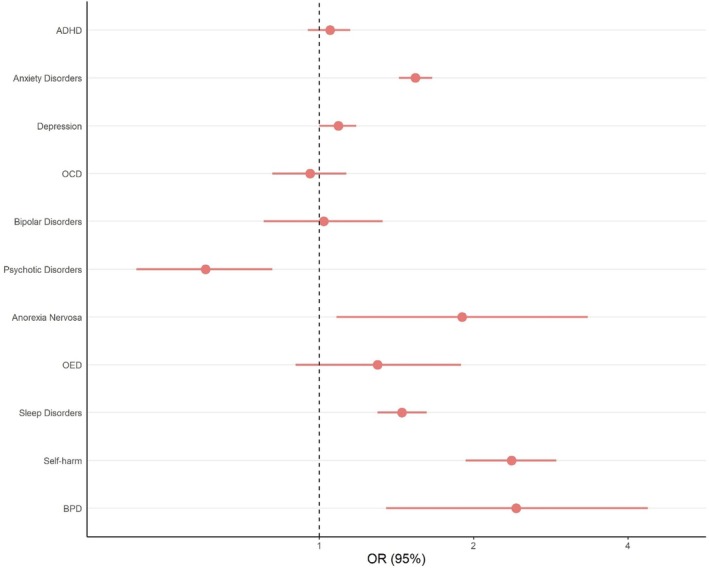
OR of retaining a preceding psychiatric diagnosis after an autism diagnosis comparing autistic males and females. Males are the reference group. ADHD, attention‐deficit hyperactivity disorder; BPD, borderline personality disorder; OCD, obsessive‐compulsive disorder; OED, other eating disorder

The birth‐year‐adjusted OR comparing the odds of another registered diagnosis/dispensed medication for the same condition between autistic females and males indicated a statistically significantly higher stability in females for anxiety, sleep disorders, self‐harm and BPD (OR_range_ = 1.45 [1.30, 1.62]–2.42 [1.35, 4.37]), and for psychotic disorders in males (OR_adjusted_ = 0.60 [0.44, 0.81], Figure 4, Table S14). In sensitivity analyses including individuals with complete 5‐year follow‐up after autism diagnosis and those diagnosed with autism before age 18, we additionally observed a statistically significantly higher stability of depression in autistic females (Table S15). Sex differences for the stability of psychotic disorders, BPD and sleep disorders were not statistically significant across sensitivity analyses. Considering sex differences in groups diagnosed with autism at different ages, we observe higher odds for stability in females for anxiety, depression and self‐harm in those diagnosed with autism between 1–8 and 9–15 (Table S16). In those receiving an autism diagnosis between ages 16–24, we additionally found higher odds for any diagnosis, ADHD and sleep disorders and decreased odds for psychotic disorders. We observed no sex differences in stability in those diagnosed with autism at age 25 and older. Sensitivity analyses considering specific time intervals between preceding and post autism diagnosis (10 years, 7 years, 4 years), consistently found higher odds for stability in females for anxiety disorders, sleep disorders and self‐harm (Table S17). Among autistic individuals with ID, anxiety (OR_adjusted_ = 2.06 [1.42, 2.99]) and self‐harm (OR_adjusted_ = 3.22 [1.54, 6.76]) were statistically significantly more stable in females compared to males (Table S18).

## Discussion

This population‐based study systematically investigated sex differences in psychiatric diagnoses preceding a diagnosis of autism and their diagnostic stability after autism diagnosis. More than 50% of autistic females and 40% of autistic males received a preceding psychiatric diagnosis, with anxiety being most common in females and ADHD in males. Autistic females showed higher odds of a preceding diagnosis for most psychiatric conditions, except psychotic disorders. Autistic males had higher odds of receiving an ADHD diagnosis prior to autism diagnosis. Notably, we observed persistent sex differences in preceding diagnoses in individuals diagnosed with autism between 2010 and 2020, despite increased autism awareness and diagnosis among females in recent years. These findings corroborate evidence from self‐report questionnaire and health‐record‐based investigations (Bölte et al., 2020; Gu et al., 2023; Kentrou et al., 2021, 2024) indicating frequent earlier contacts with specialist psychiatric care among autistic individuals, without being diagnosed with autism, particularly among females.

Preceding psychiatric diagnoses could indicate the early onset of co‐occurring psychiatric difficulties in the autistic population as a whole and females in particular. The continuously high and partly increasing probabilities of preceding psychiatric diagnoses between 2010 and 2020 observed in our sample might represent a population‐wide effect of increased mental health problems (Plana‐Ripoll et al., 2022) and care utilization (Forslund, Kosidou, Wicks, & Dalman, 2020). Moreover, they might stem from distress caused by living with undiagnosed autism and the absence of appropriate support (Camm‐Crosbie, Bradley, Shaw, Baron‐Cohen, & Cassidy, 2019; Leedham, Thompson, Smith, & Freeth, 2020; Stagg & Belcher, 2019). Persistent sex differences in preceding psychiatric diagnoses over time observed in our study underscore the importance of identifying underlying mechanisms and considering autism and sex when examining mental health (Bölte et al., 2023). While sex differences observed in this study might reflect a general trend for increased mental health problems and increased healthcare utilization in females compared to males – also observed in the nonautistic population – this is unlikely to fully explain our findings. Sex differences in this study were observed across individuals diagnosed with autism at different ages from childhood to adulthood. Moreover, sex differences in healthcare utilization in Sweden are more pronounced in primary than specialist healthcare data (Forslund et al., 2020), which were used in this study. Overall, given the common pathway of undiagnosed autistic individuals, particularly females, entering mental health services, there is a strong need for heightened awareness of potentially undiagnosed autism. Broadening education on autism and including autism screening for individuals with complex mental health needs and additionally ensuring that appropriate referrals are made (e.g. for autism assessment among individuals with mental health problems who screen positive for autism), could facilitate earlier recognition and support for autistic individuals (Mandy, 2022).

We found that sex differences in the timing of diagnosis were also largely observed in autistic individuals with preceding diagnoses. This indicates that receiving specialist psychiatric care does not necessarily facilitate the identification of autism in females. On the contrary, findings from other studies (Gu et al., 2023; Kentrou et al., 2021) suggest delayed autism assessments in those with psychiatric diagnoses. However, no causal association can be asserted from our data as a diagnosis depends on several person‐, system‐ and provider‐related factors. Nevertheless, considering increased mental health problems following later diagnosis (Hosozawa, Sacker, & Cable, 2021), our findings warrant a closer examination of referral processes for diagnostic assessment.

This study provides a novel contribution by examining the continuity of preceding psychiatric difficulties after individuals are diagnosed with autism. Importantly, our study demonstrated that the stability, defined as continued care utilization indicated through diagnosis or dispensed medication, varied considerably between psychiatric conditions. A substantial number of autistic individuals in our study re‐accessed specialist psychiatric care post autism diagnosis with the same psychiatric difficulties observed prior to autism diagnosis. This points towards persistent psychiatric difficulties warranting support (El Baou et al., 2023). Compared to autistic males, autistic females consistently showed a higher stability for anxiety, sleep disorders and self‐harm. This is consistent with previous reports (Rødgaard et al., 2021a) highlighting particularly high mental health support needs among autistic females.

Nevertheless, for most conditions about half of females and males with preceding diagnoses did not receive specialist care for these difficulties after their autism diagnosis. This could suggest improved mental health after receiving an autism diagnosis due to increased self‐compassion and self‐authenticity reported in qualitative studies (Leedham et al., 2020; Lilley et al., 2022). The instability of diagnoses might also be explained by the fluctuating or episodic nature of psychiatric symptoms and shifts between diagnoses (Oldehinkel & Ormel, 2023).

Furthermore, not retaining a diagnosis might not necessarily represent a diagnostic challenge or the absence of psychiatric difficulties. Rather it could indicate system‐level factors such as changes in the recording of diagnoses (autism being stated as the main reason for a visit) or changes in eligibility for mental health services after receiving an autism diagnosis. Previous research indicates services are less likely to accept referrals when the patient is autistic (Crane, Adams, Harper, Welch, & Pellicano, 2019). Reasons for this include insufficient training and autism knowledge (Lipinski, Boegl, Blanke, Suenkel, & Dziobek, 2022), and uncertainty as to which specialty is best suited to treat autistic individuals with mental health problems (Bölte et al., 2020; Crane et al., 2019). Future research should seek to further disentangle misdiagnosis, service access changes and barriers, to ensure adequate diagnosis and support by facilitating the navigation of the care chain and ensuring continuity and coordination between specialties (Bölte et al., 2020; Mason et al., 2021).

Diagnostic overshadowing or assigning a diagnosis based on a specific clinical picture that later proves not to be fully correct (i.e. misdiagnoses) could also contribute to the observed instability (Au‐Yeung et al., 2019; Fusar‐Poli et al., 2022). The eventual autism diagnosis may, in these cases, lead to clinical reappraisal and the dropping of a previous psychiatric diagnosis (e.g. social anxiety disorder, personality disorder) that was not accurate/optimal. Alternatively, psychiatric symptoms that genuinely persist post autism diagnosis could be overlooked or undiagnosed due to being perceived as secondary to autism. For example, self‐harm could be interpreted as an autistic repetitive behaviour (Brede et al., 2022; Cassidy, Robertson, Townsend, O'Connor, & Rodgers, 2020).

Individuals with ID showed lower odds of receiving preceding psychiatric diagnoses compared to autistic individuals without ID. This could potentially indicate a lower risk for mental health problems in autistic individuals with ID, who were found to show higher rates of premature mortality from somatic conditions, with epilepsy being the leading cause (Hirvikoski et al., 2016), indicating that somatic and neurological conditions may be more of a concern for this group than mental health conditions. However, the lower odds of psychiatric diagnoses in autistic individuals with ID could be the result of difficulties identifying psychiatric diagnoses in this group (O'Nions et al., 2024). Moreover, a diagnosis of ID could also facilitate receiving an autism diagnosis, reflected in the younger age at autism diagnosis. This could leave a shorter time span to receive other diagnoses, which however does not preclude co‐occurring mental health problems in autistic individuals with ID. The increased odds of preceding diagnoses and high stability of anxiety and depression in autistic females with ID warrant a more elaborate investigation of this group's particular mental healthcare needs.

### Strengths and limitations

The main strength of this study is the nationwide sample with follow‐up until 2020, including one of the largest samples of autistic females to date that has been followed longitudinally. Certain limitations need to be considered. We assumed a lifetime diagnosis of autism based on data from the NPR, but were unable to directly determine the long‐term validity of an autism diagnosis from the NPR data only. Our data did not capture psychiatric conditions often treated in primary care, such as depression and anxiety, or sub‐threshold symptoms. In Sweden, autism requires specialist care, meaning autistic individuals usually remain within specialist services for co‐occurring conditions, we cannot exclude that some may still receive ongoing treatment in primary care which would lead to an underestimation of stability in our analyses. Primary care data would be valuable in describing the diagnosis and care progression among autistic individuals and should be the focus of future investigations. In Sweden, both autism and psychiatric diagnoses are assessed within the mental health care system, predominantly in outpatient care for autism and both outpatient and inpatient settings for psychiatric conditions. Assessments can be conducted by the same multidisciplinary team or by separate specialized teams within mental health care. However, this is not captured in our data. This structure may influence our findings if referral bias differs between females and males. For example, if females are more likely to use healthcare services than males, they may have greater chances of being referred for certain assessments, potentially affecting the likelihood of receiving psychiatric diagnoses alongside an autism diagnosis. Outpatient care data were only available from 2001, limiting our ability to identify psychiatric difficulties requiring specialist care before that year. We used legal sex, which might not correspond to sex assigned at birth or gender identity and were unable to identify trans or non‐binary individuals, who may be more likely to experience mental health problems.

## Conclusion

This population‐based cohort study of autistic individuals systematically and robustly demonstrates that, compared to autistic males, autistic females are more likely to receive a range of psychiatric diagnoses prior to their autism diagnosis. Sex differences in preceding diagnoses are persistent despite the increased recognition of autistic females in recent years. Many preceding diagnoses required continued specialist care post autism diagnosis indicating persistent psychiatric difficulties, particularly among females. Nevertheless, a substantial number of individuals did not receive continued specialist care for their psychiatric condition after being diagnosed with autism, emphasizing the complexities of diagnosing these conditions in autistic individuals.

**Key points**

- Small studies in late‐diagnosed autistic individuals suggest that autistic individuals, particularly females, frequently receive psychiatric diagnoses before their autism diagnosis. These diagnoses might no longer be adequate after receiving an autism diagnosis.
- In a population‐based sample of autistic individuals, females persistently (2010–2020) showed increased odds for preceding psychiatric diagnoses and received autism diagnoses later than males with preceding diagnoses.
- The stability of preceding diagnoses after an autism diagnosis varied between conditions, with higher odds in females for anxiety, sleep problems and self‐harm.
- Our study suggests that autistic females are more likely to receive various psychiatric diagnoses before and after their autism diagnosis, potentially requiring continuous care. The variability in stability underscored the need for differential diagnosis in individuals with complex clinical presentations.

## Supporting information


**Table S1.** ICD and ATC codes for the included psychiatric diagnoses.
**Table S2.** Overview of sensitivity analyses.
**Table S3.** Mean age of autism diagnosis by year of diagnosis.
**Table S4.** Number of preceding diagnoses.
**Table S5.** Odds ratios of preceding diagnoses comparing males and females.
**Table S6.** Sensitivity analyses for preceding diagnoses.
**Table S7.** Prior diagnoses by autism diagnosis age groups.
**Table S8.** Proportion of preceding and stable diagnoses among autistic individuals with intellectual disability.
**Table S9.** Odds ratios of preceding diagnoses comparing autistic males and females among autistic individuals with intellectual disability.
**Table S10.** Mean age of autism diagnosis by sex and preceding diagnosis.
**Table S11.** Group differences in the mean age of autism diagnosis by sex and preceding diagnosis adjusted for birth year.
**Table S12.** Sensitivity analyses for the mean ages.
**Table S13.** Birth‐year‐adjusted mean ages among individuals with ID.
**Table S14.** Odds ratio of stability comparing males and females.
**Table S15.** Sensitivity analyses for stability.
**Table S16.** Stability of diagnoses by autism diagnosis age groups.
**Table S17.** Sensitivity analysis with different time intervals between psychiatric diagnoses prior and post autism diagnosis.
**Table S18.** Odds ratio of the stability among autistic individuals with intellectual disability.
**Figure S1.** Cohort selection process.
**Figure S2.** Proportion of autistic individuals with preceding and stable psychiatric diagnoses.
**Figure S3.** Probability of preceding diagnoses in autistic females and males diagnosed with autism from 2010 to 2020.
**Figure S4.** OR comparing preceding diagnoses between females and males diagnosed with autism between 2010 and 2020 adjusted for age at autism diagnosis.

## Data Availability

Due to Swedish data protection laws and ethical regulations, the datasets used in this study cannot be shared publicly.
