# Supplementary material for: Sex differences in psychiatric diagnoses preceding autism diagnosis and their stability post autism diagnosis
Source: J Child Psychol Psychiatry. 2025 Feb 28;66(8):1170–81. doi: 10.1111/jcpp.14130 (PMC12267683; doi:10.1111/jcpp.14130)
Supplement: Supplementary file 1 — Table S1. ICD and ATC codes for the included psychiatric diagnoses. Table S2. Overview of sensitivity analyses. Table S3. Mean age of autism diagnosis by year of diagnosis. Table S4. Number of preceding diagnoses. Table S5. Odds ratios of preceding diagnoses comparing males and females. Table S6. Sensitivity analyses for preceding diagnoses. Table S7. Prior diagnoses by autism diagnosis age groups. Table S8. Proportion of preceding and stable diagnoses among autistic individuals with intellectual disability. Table S9. Odds ratios of preceding diagnoses comparing autistic males and females among autistic individuals with intellectual disability. Table S10. Mean age of autism diagnosis by sex and preceding diagnosis. Table S11. Group differences in the mean age of autism diagnosis by sex and preceding diagnosis adjusted for birth year. Table S12. Sensitivity analyses for the mean ages. Table S13. Birth‐year‐adjusted mean ages among individuals with ID. Table S14. Odds ratio of stability comparing males and females. Table S15. Sensitivity analyses for stability. Table S16. Stability of diagnoses by autism diagnosis age groups. Table S17. Sensitivity analysis with different time intervals between psychiatric diagnoses prior and post autism diagnosis. Table S18. Odds ratio of the stability among autistic individuals with intellectual disability. Figure S1. Cohort selection process. Figure S2. Proportion of autistic individuals with preceding and stable psychiatric diagnoses. Figure S3. Probability of preceding diagnoses in autistic females and males diagnosed with autism from 2010 to 2020. Figure S4. OR comparing preceding diagnoses between females and males diagnosed with autism between 2010 and 2020 adjusted for age at autism diagnosis. [file JCPP-66-1170-s001.docx]

Sex differences in psychiatric diagnoses preceding autism diagnosis and their stability post autism diagnosis

Contents

[Table S1. ICD and ATC codes for the included psychiatric diagnoses. 2](#_Toc188020712)

[Table S2. Overview of sensitivity analyses. 3](#_Toc188020713)

[Table S3. Mean age of autism diagnosis by year of diagnosis. 4](#_Toc188020714)

[Table S4. Number of preceding diagnoses. 5](#_Toc188020715)

[Table S5. Odds ratios of preceding diagnoses comparing males and females. 6](#_Toc188020716)

[Table S6. Sensitivity analyses for preceding diagnoses. 7](#_Toc188020717)

[Table S7. Prior diagnoses by autism diagnosis age groups. 8](#_Toc188020718)

[Table S8. Proportion of preceding and stable diagnoses among autistic individuals with intellectual disability. 10](#_Toc188020719)

[Table S9. Odds ratios of preceding diagnoses comparing autistic males and females among autistic individuals with intellectual disability. 11](#_Toc188020720)

[Table S10. Mean age of autism diagnosis by sex and preceding diagnosis. 12](#_Toc188020721)

[Table S11. Group differences in the mean age of autism diagnosis by sex and preceding diagnosis adjusted for birth year. 13](#_Toc188020722)

[Table S12. Sensitivity analyses for the mean ages. 14](#_Toc188020723)

[Table S13. Birth-year adjusted mean ages among individuals with ID. 15](#_Toc188020724)

[Table S14. Odds ratio of stability comparing males and females. 16](#_Toc188020725)

[Table S15. Sensitivity analyses for stability. 17](#_Toc188020726)

[Table S16. Stability of diagnoses by autism diagnosis age groups. 18](#_Toc188020727)

[Table S17. Sensitivity analysis with different time intervals between psychiatric diagnoses prior and post-autism diagnosis. 20](#_Toc188020728)

[Table S18. Odds ratio of the stability among autistic individuals with intellectual disability. 21](#_Toc188020729)

[Figure S1. Cohort selection process. 22](#_Toc188020730)

[Figure S2. Proportion of autistic individuals with preceding and stable psychiatric diagnoses. 23](#_Toc188020731)

[Figure S3. Probability of preceding diagnoses in autistic females and males diagnosed with autism from 2010 to 2020. 24](#_Toc188020732)

[Figure S4. OR comparing preceding diagnoses between females and males diagnosed with autism between 2010-2020 adjusted for age at autism diagnosis. 25](#_Toc188020733)

# Table S1. ICD and ATC codes for the included psychiatric diagnoses.

| **Type of mental health problem** | **Classification** | **Diagnostic/**  **ACT code** | **Diagnosis/Drug** |
| --- | --- | --- | --- |
|  | | | |
| Anxiety disorders | ICD-9 | 300A  300C | Anxiety states  Phobic disorders |
|  | ICD-10 | F40  F41 | Phobic anxiety disorders  Other anxiety disorders |
| Attention Deficit Hyperactivity Disorder (ADHD) | ICD-9 | 314 | Hyperkinetic syndrome of childhood |
|  | ICD-10 | F90 | Hyperkinetic disorders |
|  | ATC | N06BA04  N06BA01  N06BA02  N06BA09  N06BA12 | Methylphenidate  Amphetamine  Dexamphetamine  Atomoxetine  Lisdexamfetamine |
| Depressive disorders | ICD-9 | 296B  300E  311 | Major depressive disorder single episode  Dysthymic disorder  Depressive disorder, not elsewhere classified |
|  | ICD-10 | F32  F33  F34 (excluding 34.0)  F38  F39 | Depressive episode  Recurrent depressive disorder  Persistent mood disorders  Other mood (affective) disorders  Unspecified mood (affective) disorder |
| Obsessive compulsive disorder (OCD) | ICD-9 | 300D | Obsessive-compulsive disorders |
|  | ICD-10 | F42 | Obsessive-compulsive disorder |
| Bipolar disorders | ICD-9 | 296 (excluding 296B) | Affective Psychosis |
|  | ICD-10 | F30  F31  F34.0 | Manic episode  Bipolar affective disorder  Cyclothymia |
|  | ATC | N05AN01 | Lithium |
| Psychotic disorders | ICD-9 | 295  297  298 | Schizophrenic disorders  Delusional disorders  Other nonorganic psychoses |
|  | ICD-10 | F20-29 | Schizophrenia, schizotypal and delusional diosrders |
|  | ATC | N05AH02 | Clozapine |
| Anorexia nervosa | ICD-9 | 307B | Anorexia nervosa |
|  | ICD-10 | F50.0  F50.1 | Anorexia nervosa  Atypical anorexia nervosa |
| Other eating disorders | ICD-10 | F50.9  F 50.2  F 50.3 | Eating disorder, unspecified  Bulimia nervosa  Bulimia nervosa, unspecified |
| Borderline personality disorder (BPD) | ICD-10 | F60.3 | Emotionally unstable personality disorder |
| Sleep disorders | ICD-9 | 307E  780F | Specific disorders of sleep of nonorganic origin  Sleep disturbances |
|  | ICD-10 | F51  G47 | Nonorganic sleep disorders  Sleep disorders |
|  | ATC | N05CF01  N05CF02  N05CF03 | Zopiclone  Zolpidem  Zaleplon |
| Self-harm | ICD-9 | E950-E959  E980-E989 | Suicide And Self-Inflicted Injury  Injury Undetermined Whether Accidentally Or Purposely Inflicted |
|  | ICD-10 | X60-X84  Y10-Y34 | Intentional self-harm  Event of undetermined intent |
| Intellectual Disability | ICD-9 | 317-319 | Intellectual disabilities |
|  | ICD-10 | F70-F79 | Mental retardation |

# Table S2. Overview of sensitivity analyses.

| Sensitivity analysis | Explanation | Purpose |
| --- | --- | --- |
| S1 | Repeating the analysis on preceding diagnoses in individuals with complete 2- and 5-year coverage prior to autism diagnosis. | Test the effect of differing lengths of follow-up. |
| S2 | Repeating the stability analyses in individuals with complete follow-up for 5 years after autism diagnosis. | Test the effect of differing lengths of follow-up. |
| S3 | Repeat the stability analyses in individuals that received the psychiatric diagnosis at least twice after autism diagnosis. | To reduce diagnostic uncertainty. |
| S4* | Repeat analyses in a sample restricted to those diagnosed with autism before age 18. | To account for procedural differences and challenges in establishing a diagnosis of autism in adulthood and the longer follow-up time to receive a preceding diagnosis in adult-diagnosed autistic individuals. |
| S5* | Repeat analyses in a sample that only includes individuals born after 2000. | To account for the fact that ICD-10 codes for borderline personality disorder (BPD) and certain eating disorders in the ‘other eating disorders’ category were only available after 1997 and data from outpatient care were only available from 2001 onwards. |
| S6 | Repeat analyses in different groups based on the age of autism diagnosis (1-8, 9-15, 16-24,25+). | To account for varying age of onset and prevalence rates of psychiatric diagnoses. |
| S7 | Repeats the stability analysis with different intervals between the psychiatric diagnosis preceding and following an autism diagnosis | To account for different lengths between the psychiatric diagnoses prior and post autism diagnosis. |
| *Note.* In the models adjusted for birth year and age at autism diagnosis we used 5 splines for S4 and 3 splines for all other sensitivity analyses. The FDR threshold for the sensitivity analyses was p=.005.  *** Analyses S4 and S5 did not include any results for BPD as there were no males with a diagnosis. | | |

# Table S3. Mean age of autism diagnosis by year of diagnosis.

| **Diagnosis year** | **Mean – overall** | **SD – overall** | **Mean – females** | **SD – females** | **Mean – males** | **SD – males** |
| --- | --- | --- | --- | --- | --- | --- |
| 1991 | 1.35 | - | 1.35 | - | - | - |
| 1992 | 2.14 | 0.59 | 2.59 | - | 2.06 | 0.61 |
| 1993 | 2.99 | 0.52 | 2.61 | 0.39 | 3.15 | 0.49 |
| 1994 | 3.41 | 0.94 | 3.76 | 1.08 | 3.32 | 0.89 |
| 1995 | 4.05 | 1.04 | 4.07 | 0.89 | 4.04 | 1.09 |
| 1996 | 4.23 | 1.10 | 3.69 | 0.99 | 4.34 | 1.10 |
| 1997 | 5.16 | 1.59 | 4.97 | 1.72 | 5.28 | 1.52 |
| 1998 | 5.03 | 1.61 | 4.67 | 1.91 | 5.10 | 1.55 |
| 1999 | 5.58 | 1.90 | 5.70 | 2.43 | 5.54 | 1.75 |
| 2000 | 6.57 | 2.35 | 6.64 | 1.93 | 6.55 | 2.49 |
| 2001 | 7.45 | 2.46 | 7.37 | 2.50 | 7.48 | 2.45 |
| 2002 | 8.01 | 2.83 | 8.14 | 2.86 | 7.97 | 2.82 |
| 2003 | 8.59 | 3.20 | 8.68 | 3.35 | 8.56 | 3.15 |
| 2004 | 8.98 | 3.44 | 8.83 | 3.55 | 9.02 | 3.41 |
| 2005 | 9.80 | 3.90 | 10.33 | 3.96 | 9.62 | 3.87 |
| 2006 | 10.33 | 3.99 | 11.18 | 3.95 | 10.04 | 3.96 |
| 2007 | 11.11 | 4.28 | 11.96 | 4.23 | 10.78 | 4.26 |
| 2008 | 11.73 | 4.56 | 12.99 | 4.42 | 11.24 | 4.52 |
| 2009 | 11.82 | 4.86 | 13.10 | 4.63 | 11.30 | 4.86 |
| 2010 | 12.37 | 5.20 | 13.37 | 5.14 | 11.97 | 5.16 |
| 2011 | 12.56 | 5.19 | 13.98 | 4.98 | 11.93 | 5.15 |
| 2012 | 12.81 | 5.28 | 14.05 | 5.24 | 12.27 | 5.21 |
| 2013 | 12.81 | 5.58 | 14.42 | 5.31 | 12.10 | 5.55 |
| 2014 | 13.23 | 5.85 | 14.63 | 5.63 | 12.57 | 5.84 |
| 2015 | 13.17 | 6.06 | 14.75 | 5.75 | 12.42 | 6.06 |
| 2016 | 13.35 | 6.34 | 14.81 | 6.02 | 12.63 | 6.37 |
| 2017 | 13.35 | 6.32 | 14.97 | 6.00 | 12.47 | 6.32 |
| 2018 | 13.84 | 6.50 | 15.40 | 6.18 | 12.91 | 6.51 |
| 2019 | 14.30 | 6.49 | 15.78 | 6.08 | 13.36 | 6.57 |
| 2020 | 14.92 | 6.32 | 16.43 | 5.82 | 13.85 | 6.44 |

# Table S4. Number of preceding diagnoses.

|  | **Overall** | **Autistic males** | **Autistic females** |
| --- | --- | --- | --- |
|  |  |  |  |
| **N (%)** | 72,331 | 48,221 | 24,110 |
|  |  |  |  |
| **Number of diagnoses** |  |  |  |
| 0 | 39,557 (54.7) | 28,521 (59.1) | 11,036 (45.8) |
| 1 | 19,693 (27.2) | 13,452 (27.9) | 6,241 (25.9) |
| 2 | 7,470 (10.3) | 4,054 (8.4) | 3,416 (14.2) |
| 3 | 3,336 (4.6) | 1,480 (3.1) | 1,856 (7.7) |
| 4 | 1,357 (1.9) | 486 (1.0) | 871 (3.6) |
| 5 | 546 (0.8) | 159 (0.3) | 387 (1.6) |
| 6 | 224 (0.3) | 47 (0.1) | 177 (0.7) |
| 7+ | 148 (0.2) | 22 (0.0) | 126 (0.5) |
|  |  |  |  |
|  |  |  |  |

# Table S5. Odds ratios of preceding diagnoses comparing males and females.

| N= 72 331 | **OR** | **lower CI** | **upper CI** | **p** |
| --- | --- | --- | --- | --- |
| **Crude model** | | | | |
| Any Disorder | 1.72 | 1.66 | 1.77 | <.001 |
| ADHD | 0.86 | 0.83 | 0.89 | <.001 |
| Anxiety Disorders | 3.45 | 3.31 | 3.60 | <.001 |
| Depressive Disorders | 2.64 | 2.53 | 2.76 | <.001 |
| Obsessive-Compulsive Disorder | 1.83 | 1.68 | 1.99 | <.001 |
| Bipolar Disorders | 2.87 | 2.51 | 3.28 | <.001 |
| Psychotic Disorders | 1.38 | 1.19 | 1.60 | <.001 |
| Anorexia Nervosa | 13.88 | 10.85 | 17.76 | <.001 |
| Other Eating Disorders | 9.97 | 8.55 | 11.63 | <.001 |
| Sleep Disorders | 1.88 | 1.78 | 1.98 | <.001 |
| Self-harm | 3.27 | 3.02 | 3.54 | <.001 |
| Borderline Personality Disorder | 15.17 | 11.48 | 20.06 | <.001 |
| **Adjusted for birth year and age of autism diagnosis** | | | | |
| Any Disorder | 1.18 | 1.14 | 1.22 | <.001 |
| ADHD | 0.69 | 0.66 | 0.71 | <.001 |
| Anxiety Disorders | 2.48 | 2.37 | 2.60 | <.001 |
| Depressive Disorders | 1.76 | 1.68 | 1.85 | <.001 |
| Obsessive-Compulsive Disorder | 1.29 | 1.18 | 1.41 | <.001 |
| Bipolar Disorders | 1.96 | 1.72 | 2.25 | <.001 |
| Psychotic Disorders | 0.91 | 0.78 | 1.06 | 0.229 |
| Anorexia Nervosa | 9.07 | 7.11 | 11.58 | <.001 |
| Other Eating Disorders | 6.85 | 5.88 | 7.98 | <.001 |
| Sleep Disorders | 1.46 | 1.38 | 1.54 | <.001 |
| Self-harm | 2.36 | 2.18 | 2.56 | <.001 |
| Borderline Personality Disorder | 10.69 | 8.06 | 14.17 | <.001 |
| *Note.* Males are the reference category. Abbreviations: ADHD – attention deficit hyperactivity disorder. | | | | |

# Table S6. Sensitivity analyses for preceding diagnoses.

| **Sensitivity analysis** | **S1^a^** | | | | **S4^b^** | | **S5^c^** | |
| --- | --- | --- | --- | --- | --- | --- | --- | --- |
| **N** | N= 64,891 | | | | N= 58,152 | | N= 39,513 | |
|  | **5 years** | | **2 years** | |  | |  | |
|  | **OR [95% CI]** | **p** | **OR [95% CI]** | **p** | **OR [95% CI]** | **p** | **OR [95% CI]** | **p** |
|  | | | | |  |  |  |  |
| **Adjusted for birth year and age of autism diagnosis** | | | | |  |  |  |  |
| Any Disorder | 1.20 [1.16, 1.24] | <.001 | 1.21 [1.17,1.25] | <.001 | 1.11 [1.06, 1.15] | <.001 | 1.13 [1.08, 1.19] | <.001 |
| ADHD | 0.70 [0.67, 0.72] | <.001 | 0.72 [0.69, 0.75] | <.001 | 0.66 [0.63, 0.69] | <.001 | 0.68 [0.64, 0.72] | <.001 |
| Anxiety Disorders | 2.48 [2.37, 2.59] | <.001 | 2.43 [2.32, 2.55] | <.001 | 2.51 [2.36, 2.66] | <.001 | 2.60 [2.42, 2.80] | <.001 |
| Depressive Disorders | 1.73 [1.65, 1.81] | <.001 | 1.69 [1.61, 1.78] | <.001 | 1.95 [1.82, 2.08] | <.001 | 2.00 [1.83, 2.19] | <.001 |
| OCD | 1.29 [1.18, 1.42] | <.001 | 1.29 [1.17, 1.42] | <.001 | 1.26 [1.12, 1.42] | <.001 | 1.25 [1.07, 1.45] | 0.004 |
| Bipolar Disorders | 1.92 [1.67, 2.21] | <.001 | 1.96 [1.68, 2.28] | <.001 | 1.70 [1.32, 2.18] | <.001 | 1.48 [1.02, 2.15] | 0.037 |
| Psychotic Disorders | 0.90 [0.77, 1.05] | 0.191 | 0.84 [0.71, 1.00] | 0.053 | 1.53 [1.13, 2.07] | 0.006 | 1.49 [0.88, 2.52] | 0.14 |
| Anorexia Nervosa | 9.84 [7.58, 12.79] | <.001 | 10.43 [7.70, 14.12] | <.001 | 7.85 [5.75, 10.72] | <.001 | 8.18 [5.30, 12.62] | <.001 |
| Other Eating Disorders | 7.02 [5.95, 8.27] | <.001 | 7.38 [6.10, 8.92] | <.001 | 5.15 [4.23, 6.28] | <.001 | 5.05 [3.93, 6.50] | <.001 |
| Sleep Disorders | 1.53 [1.44, 1.62] | <.001 | 1.54 [1.44, 1.64] | <.001 | 1.36 [1.26, 1.47] | <.001 | 1.33 [1.22, 1.46] | <.001 |
| Self-harm | 2.99 [2.72, 3.28] | <.001 | 3.15 [2.82, 3.53] | <.001 | 2.24 [2.00, 2.51] | <.001 | 1.82 [1.58, 2.11] | <.001 |
| BPD | 11.05 [8.26, 14.80] | <.001 | 11.04 [8.04, 15.14] | <.001 | * | * | * | * |
| *Note.* Males are the reference category. Abbreviations: ADHD – attention deficit hyperactivity disorder, OCD – obsessive-compulsive disorder, BPD – borderline personality disorder.  * No results are shown for borderline personality disorder due to sample size. ^a^ Preceding diagnoses in individuals with complete 2 and 5 year follow-up prior to autism diagnosis.  ^b^ Includes only those diagnosed with autism before age 18.  ^c^ Includes only individuals born after 2000. | | | | | | | | |
|  | | | | |  |  |  |  |

# Table S7. Prior diagnoses by autism diagnosis age groups.

|  | **OR** | **lower CI** | **upper CI** | **p** |
| --- | --- | --- | --- | --- |
| **Autism diagnosis age 1-8** (N= 20 275) | | | | |
| Any Disorder | 1.01 | 0.91 | 1.11 | 0.895 |
| ADHD | 0.84 | 0.75 | 0.95 | 0.005 |
| Anxiety Disorders | 2.37 | 1.65 | 3.41 | <.001 |
| Depressive Disorders | 1.82 | 0.73 | 4.54 | 0.197 |
| Obsessive-Compulsive Disorder | 1.63 | 0.86 | 3.08 | 0.131 |
| Bipolar Disorders | * | * | * | * |
| Psychotic Disorders | * | * | * | * |
| Anorexia Nervosa | * | * | * | * |
| Other Eating Disorders | 0.91 | 0.37 | 2.22 | 0.83 |
| Sleep Disorders | 1.38 | 1.17 | 1.63 | <.001 |
| Self-harm | 0.94 | 0.60 | 1.46 | 0.768 |
| Borderline Personality Disorder | * | * | * | * |
| **Autism diagnosis age 9-15** (N= 29 922) | | | | |
| Any Disorder | 1.05 | 0.99 | 1.10 | 0.08 |
| ADHD | 0.63 | 0.60 | 0.66 | <.001 |
| Anxiety Disorders | 2.26 | 2.10 | 2.43 | <.001 |
| Depressive Disorders | 1.97 | 1.81 | 2.15 | <.001 |
| Obsessive-Compulsive Disorder | 1.32 | 1.15 | 1.53 | <.001 |
| Bipolar Disorders | 1.41 | 1.01 | 1.97 | 0.044 |
| Psychotic Disorders | 2.63 | 1.70 | 4.06 | <.001 |
| Anorexia Nervosa | 7.84 | 5.19 | 11.83 | <.001 |
| Other Eating Disorders | 6.17 | 4.61 | 8.24 | <.001 |
| Sleep Disorders | 1.28 | 1.16 | 1.42 | <.001 |
| Self-harm | 1.96 | 1.68 | 2.29 | <.001 |
| Borderline Personality Disorder | * | * | * | * |
| **Autism diagnosis age 16-24** (N= 19 559) | | | | |
| Any Disorder | 1.46 | 1.37 | 1.56 | <.001 |
| ADHD | 0.72 | 0.67 | 0.76 | <.001 |
| Anxiety Disorders | 2.71 | 2.54 | 2.89 | <.001 |
| Depressive Disorders | 1.72 | 1.62 | 1.84 | <.001 |
| Obsessive-Compulsive Disorder | 1.29 | 1.14 | 1.46 | <.001 |
| Bipolar Disorders | 1.95 | 1.64 | 2.32 | <.001 |
| Psychotic Disorders | 0.82 | 0.68 | 0.99 | 0.039 |
| Anorexia Nervosa | 9.93 | 7.03 | 14.02 | <.001 |
| Other Eating Disorders | 7.48 | 6.04 | 9.25 | <.001 |
| Sleep Disorders | 1.60 | 1.47 | 1.73 | <.001 |
| Self-harm | 2.83 | 2.54 | 3.16 | <.001 |
| Borderline Personality Disorder | 13.75 | 9.35 | 20.22 | <.001 |
| **Autism diagnosis age 25 and older** (N= 2575) | | | | |
| Any Disorder | 1.70 | 1.39 | 2.08 | <.001 |
| ADHD | 0.90 | 0.76 | 1.07 | 0.242 |
| Anxiety Disorders | 2.29 | 1.95 | 2.69 | <.001 |
| Depressive Disorders | 1.40 | 1.20 | 1.64 | <.001 |
| Obsessive-Compulsive Disorder | 1.11 | 0.82 | 1.50 | 0.487 |
| Bipolar Disorders | 2.6 | 1.95 | 3.48 | <.001 |
| Psychotic Disorders | 0.71 | 0.51 | 0.97 | 0.034 |
| Anorexia Nervosa | 12.15 | 5.50 | 26.81 | <.001 |
| Other Eating Disorders | 12.73 | 7.40 | 21.89 | <.001 |
| Sleep Disorders | 1.53 | 1.30 | 1.81 | <.001 |
| Self-harm | 2.13 | 1.68 | 2.70 | <.001 |
| Borderline Personality Disorder | 7.14 | 4.69 | 10.88 | <.001 |
| *Note.* Males are the reference category. Abbreviations: ADHD – attention deficit hyperactivity disorder. *Not calculated as there were less than 5 individuals in each group. | | | | |

# Table S8. Proportion of preceding and stable diagnoses among autistic individuals with intellectual disability.

| N= 10,331 | **Overall** | **Autistic males** | **Autistic females** | **p** |  |
| --- | --- | --- | --- | --- | --- |
| **Preceding psychiatric diagnosis: N (%)** | | | | | |
| N | 10,331 | 7,277 | 3,054 |  |  |
| Any Disorder | 2,741 (26.5) | 1,836 (25.2) | 905 (29.6) | <.001 |  |
| ADHD | 1,997 (19.3) | 1,414 (19.4) | 583 (19.1) | 0.709 |  |
| Anxiety Disorders | 483 (4.7) | 212 (2.9) | 271 (8.9) | <.001 |  |
| Depressive Disorders | 304 (2.9) | 136 (1.9) | 168 (5.5) | <.001 |  |
| Obsessive-Compulsive Disorder | 111 (1.1) | 71 (1.0) | 40 (1.3) | 0.162 |  |
| Bipolar Disorders | 61 (0.6) | 23 (0.3) | 38 (1.2) | <.001 |  |
| Psychotic Disorders | 101 (1.0) | 55 (0.8) | 46 (1.5) | 0.001 |  |
| Anorexia Nervosa |  | < 5 | 19 (0.6) | <.001 |  |
| Other Eating Disorders | 59 (0.6) | 16 (0.2) | 43 (1.4) | <.001 |  |
| Sleep Disorders | 500 (4.8) | 320 (4.4) | 180 (5.9) | 0.001 |  |
| Self-harm | 209 (2.0) | 95 (1.3) | 114 (3.7) | <.001 |  |
| Borderline Personality Disorder |  | < 5 | 23 (0.8) | <.001 |  |
| **Stable psychiatric diagnosis among those with a prior diagnosis: N (%)** | | | | | |
| ADHD | 1,678 (84.0) | 1,192 (84.4) | 486 (83.4) | 0.577 |  |
| Anxiety Disorders | 232 (48.0) | 79 (37.3) | 153 (56.5) | <.001 |  |
| Depressive Disorders | 134 (44.1) | 49 (36.0) | 85 (50.6) | <.001 |  |
| Obsessive-Compulsive Disorder | 60 (54.1) | 39 (54.9) | 21 (52.5) | 0.433 |  |
| Bipolar Disorders | 38 (62.3) | 14 (60.9) | 24 (63.2) | <.001 |  |
| Psychotic Disorders | 48 (47.5) | 28 (50.9) | 20 (43.5) | 0.092 |  |
| Anorexia Nervosa |  | 0 | < 5 | 0.011 |  |
| Other Eating Disorders |  | < 5 | 17 (39.5) | <.001 |  |
| Sleep Disorders | 161 (32.2) | 86 (26.9) | 75 (41.7) | <.001 |  |
| Self-harm | 56 (26.8) | 13 (13.7) | 43 (37.7) | <.001 |  |
| Borderline Personality Disorder | 18 (69.2) | 0 (0.0) | 18 (78.3) | <.001 |  |
| *Note.* Males are the reference category. Abbreviations: ADHD – attention deficit hyperactivity. | | | | |  |

# Table S9. Odds ratios of preceding diagnoses comparing autistic males and females among autistic individuals with intellectual disability.

| N= 10,331 | **OR** | **lower CI** | **upper CI** | **p** |
| --- | --- | --- | --- | --- |
| **Crude model** | | | | |
| Any Disorder | 1.25 | 1.14 | 1.37 | <.001 |
| ADHD | 0.98 | 0.88 | 1.09 | 0.690 |
| Anxiety Disorders | 3.25 | 2.69 | 3.91 | <.001 |
| Depressive Disorders | 3.06 | 2.43 | 3.85 | <.001 |
| Obsessive-Compulsive Disorder | 1.35 | 0.91 | 1.99 | 0.134 |
| Bipolar Disorders | 3.97 | 2.36 | 6.68 | <.001 |
| Psychotic Disorders | 2.01 | 1.35 | 2.98 | 0.001 |
| Anorexia Nervosa | 11.38 | 3.87 | 33.49 | <.001 |
| Other Eating Disorders | 6.48 | 3.64 | 11.52 | <.001 |
| Sleep Disorders | 1.36 | 1.13 | 1.64 | 0.001 |
| Self-harm | 2.93 | 2.23 | 3.86 | <.001 |
| Borderline Personality Disorder | 18.40 | 5.52 | 61.33 | <.001 |
| **Adjusted for birth year and age of autism diagnosis** | | | | |
| Any Disorder | 0.92 | 0.82 | 1.02 | 0.130 |
| ADHD | 0.73 | 0.64 | 0.82 | <.001 |
| Anxiety Disorders | 2.39 | 1.95 | 2.92 | <.001 |
| Depressive Disorders | 2.07 | 1.63 | 2.64 | <.001 |
| Obsessive-Compulsive Disorder | 0.93 | 0.62 | 1.38 | 0.703 |
| Bipolar Disorders | 2.62 | 1.56 | 4.40 | <.001 |
| Psychotic Disorders | 1.26 | 0.83 | 1.90 | 0.282 |
| Anorexia Nervosa | 7.82 | 2.76 | 22.13 | <.001 |
| Other Eating Disorders | 4.83 | 2.75 | 8.46 | <.001 |
| Sleep Disorders | 1.09 | 0.90 | 1.33 | 0.380 |
| Self-harm | 2.15 | 1.63 | 2.84 | <.001 |
| Borderline Personality Disorder | 10.82 | 3.20 | 36.59 | <.001 |
| *Note.* Males are the reference category. Abbreviations: ADHD – attention deficit hyperactivity disorder. | | | | |

# Table S10. Mean age of autism diagnosis by sex and preceding diagnosis.

|  | **Overall** | | **Autistic males** | | **Autistic females** | |
| --- | --- | --- | --- | --- | --- | --- |
| **Disorder** | **without** | **with** | **without** | **with** | **without** | **with** |
| Any Disorder | 10.59 [5.49] | 15.93 [5.27] | 10.14 [5.37] | 15.22 [5.38] | 11.77 [5.61] | 17.00 [4.91] |
| ADHD | 12.41 [6.27] | 14.69 [4.84] | 11.49 [6.16] | 14.15 [4.75] | 14.19 [6.09] | 15.90 [4.83] |
| Anxiety Disorders | 11.99 [5.64] | 18.60 [4.78] | 11.54 [5.59] | 18.55 [5.14] | 13.11 [5.58] | 18.64 [4.50] |
| Depressive Disorders | 12.04 [5.62] | 19.33 [4.42] | 11.46 [5.52] | 19.51 [4.62] | 13.36 [5.61] | 19.16 [4.24] |
| OCD | 12.87 [5.99] | 17.51 [4.75] | 12.10 [5.90] | 17.20 [4.91] | 14.46 [5.86] | 17.86 [4.55] |
| Bipolar Disorders | 12.91 [5.95] | 21.21 [4.79] | 12.15 [5.89] | 20.54 [5.05] | 14.44 [5.78] | 21.69 [4.54] |
| Psychotic Disorders | 12.92 [5.96] | 21.32 [4.29] | 12.13 [5.87] | 21.76 [4.10] | 14.53 [5.83] | 20.67 [4.48] |
| Anorexia Nervosa | 12.97 [6.00] | 18.72 [4.32] | 12.21 [5.92] | 17.58 [5.09] | 14.51 [5.85] | 18.88 [4.18] |
| OED | 12.92 [5.98] | 18.88 [4.85] | 12.20 [5.92] | 16.56 [5.96] | 14.41 [5.82] | 19.37 [4.44] |
| Sleep Disorders | 12.56 [5.77] | 17.65 [6.43] | 11.87 [5.71] | 16.72 [6.76] | 14.02 [5.61] | 18.69 [5.85] |
| Self-harm | 12.79 [5.93] | 18.59 [5.22] | 12.09 [5.86] | 17.75 [6.09] | 14.27 [5.80] | 19.13 [4.50] |
| BPD | 12.94 [5.96] | 23.63 [3.12] | 12.20 [5.91] | 24.39 [3.04] | 14.45 [5.77] | 23.53 [3.12] |
| *Note.* This table shows the mean age [standard deviation] of autism diagnosis across preceding diagnoses by sex. Abbreviations: ADHD – attention deficit hyperactivity disorder, OCD – obsessive-compulsive disorder, OED – other eating disorders, BPD – borderline personality disorder. | | | | | | |

# Table S11. Group differences in the mean age of autism diagnosis by sex and preceding diagnosis adjusted for birth year.

|  | **Mean ages [standard errors] of diagnosis adjusted for birth year** | | | | **Group comparisons** | | | | | | | |
| --- | --- | --- | --- | --- | --- | --- | --- | --- | --- | --- | --- | --- |
| **Diagnosis** | Males without a preceding diagnosis (M) | Males with a preceding diagnosis (M+) | Females without a preceding diagnosis (F) | Females with a preceding diagnosis (F+) | MM+ | P_MM+_ | MF | P_MF_ | FF+ | P_FF+_ | M+F+ | P_M+F+_ |
| Any diagnosis | 11.80 [0.08] | 14.92 [0.04] | 12.79 [0.05] | 16.19 [0.04] | -3.118 | <.001 | -0.986 | <.001 | -3.400 | <.001 | -1.268 | <.001 |
| ADHD | 12.89 [0.04] | 14.44 [0.04] | 14.50 [0.04] | 15.65 [0.05] | -1.546 | <.001 | -1.610 | <.001 | -1.156 | <.001 | -1.22 | <.001 |
| Anxiety Disorders | 12.85 [0.04] | 17.12 [0.05] | 13.81 [0.04] | 17.41 [0.04] | -4.261 | <.001 | -0.951 | <.001 | -3.609 | <.001 | -0.299 | <.001 |
| Depressive Disorders | 12.88 [0.04] | 17.45 [0.06] | 14.09 [0.04] | 17.52 [0.05] | -4.574 | <.001 | -1.212 | <.001 | -3.431 | <.001 | -0.069 | 0.264 |
| OCD | 13.35 [0.04] | 16.08 [0.10] | 14.79 [0.04] | 16.72 [0.10] | -2.728 | <.001 | -1.434 | <.001 | -1.936 | <.001 | -0.643 | <.001 |
| Bipolar Disorder | 13.42 [0.04] | 17.38 [0.19] | 14.82 [0.04] | 18.48 [0.14] | -3.960 | <.001 | -1.401 | <.001 | -3.661 | <.001 | -1.102 | <.001 |
| Psychotic Disorders | 13.41 [0.04] | 18.12 [0.16] | 14.87 [0.04] | 17.65 [0.19] | -4.710 | <.001 | -1.459 | <.001 | -2.78 | <.001 | 0.471 | 0.049 |
| Anorexia Nervosa | 13.43 [0.04] | 15.98 [0.47] | 14.83 [0.04] | 17.25 [0.13] | -2.550 | <.001 | -1.400 | <.001 | -2.425 | <.001 | -1.275 | 0.008 |
| OED | 13.42 [0.04] | 16.16 [0.24] | 14.77 [0.04] | 17.51 [0.10] | -2.739 | <.001 | -1.351 | <.001 | -2.744 | <.001 | -1.357 | <.001 |
| Sleep Disorders | 13.27 [0.04] | 16.30 [0.07] | 14.60 [0.04] | 17.31 [0.07] | -3.031 | <.001 | -1.337 | <.001 | -2.702 | <.001 | -1.008 | <.001 |
| Self-harm | 13.37 [0.04] | 16.32 [0.12] | 14.73 [0.04] | 17.07 [0.08] | -2.947 | <.001 | -1.358 | <.001 | -2.341 | <.001 | -0.751 | <.001 |
| BPD | 13.45 [0.04] | 19.74 [0.36] | 14.83 [0.04] | 19.38 [0.14] | -6.293 | <.001 | -1.377 | <.001 | -4.550 | <.001 | 0.367 | 0.333 |
| *Note.* This table compares the birth year adjusted (reference year: 2000) mean age at autism diagnoses between autistic males with and without a preceding diagnosis (MM+), autistic males and females (MF), autistic females with and without a preceding diagnosis (FF+), and autistic males and females with a preceding diagnosis (M+F+). Abbreviations: ADHD – attention deficit hyperactivity disorder, OCD – obsessive-compulsive disorder, OED – other eating disorders, BPD – borderline personality disorder. | | | | | | | | | | | | |

# Table S12. Sensitivity analyses for the mean ages.

|  | **Mean ages [standard errors] of diagnosis adjusted for birth year** | | | | **Group comparisons** | | | | | | | |
| --- | --- | --- | --- | --- | --- | --- | --- | --- | --- | --- | --- | --- |
| Diagnosis | Males without a preceding diagnosis (M) | Males with a preceding diagnosis (M+) | Females without a preceding diagnosis (F) | Females with a preceding diagnosis (F) | MM+ | P_MM+_ | MF | P_MF_ | FF+ | P_FF+_ | M+F+ | P_M+F+_ |
| **Sensitivity analysis including only those diagnosed with autism before age 18** | | | | | | | | | | | | |
| Any diagnosis | 11.52 [0.04] | 13.82 [0.03] | 12.40 [0.05] | 15.04 [0.03] | -2,302 | <.001 | -0,885 | <.001 | -2,634 | <.001 | -1,217 | <0.001 |
| ADHD | 12.08 [0.04] | 13.74 [0.03] | 13.49 [0.04] | 14.68 [0.04] | -1,655 | <.001 | -1,411 | <.001 | -1,186 | <.001 | -0,942 | <0.001 |
| Anxiety Disorders | 12.32 [0.03] | 15.20 [0.05] | 13.22 [0.04] | 15.96 [0.03] | -2,883 | <.001 | -0,903 | <.001 | -2,739 | <.001 | -0,759 | <0.001 |
| Depressive Disorders | 12.34 [0.03] | 15.65 [0.05] | 13.37 [0.04] | 16.13 [0.03] | -3,31 | <.001 | -1,028 | <.001 | -2,764 | <.001 | -0,482 | <0.001 |
| OCD | 12.58 [0.03] | 14.98 [0.08] | 13.79 [0.04] | 15.60 [0.08] | -2,397 | <.001 | -1,211 | <.001 | -1,806 | <.001 | -0,62 | <0.001 |
| Bipolar Disorder | 12.64 [0.03] | 14.64 [0.22] | 13.85 [0.04] | 16.00 [0.14] | -2,001 | <.001 | -1,213 | <.001 | -2,144 | <.001 | -1,356 | <0.001 |
| Psychotic Disorders | 12.64 [0.03] | 16.04 [0.18] | 13.86 [0.04] | 15.78 [0.16] | -3,398 | <.001 | -1,220 | <.001 | -1,921 | <.001 | 0,257 | 0.286 |
| Anorexia Nervosa | 12.64 [0.03] | 15.19 [0.40] | 13.83 [0.04] | 16.29 [0.08] | -2,551 | <.001 | -1,190 | <.001 | -2,458 | <.001 | -1,097 | 0.008 |
| OED | 12.63 [0.03] | 14.82 [0.23] | 13.80 [0.04] | 16.15 [0.08] | -2,187 | <.001 | -1,174 | <.001 | -2,349 | <.001 | -1,335 | <0.001 |
| Sleep Disorders | 12.58 [0.03] | 14.04 [0.07] | 13.76 [0.04] | 15.14 [0.07] | -1,459 | <.001 | -1,187 | <.001 | -1,382 | <.001 | -1,109 | <0.001 |
| Self-harm | 12.61 [0.03] | 14.34 [0.12] | 13.77 [0.04] | 15.87 [0.07] | -1,728 | <.001 | -1,161 | <.001 | -2,094 | <.001 | -1,527 | <0.001 |
| **Sensitivity analysis including only individuals born after 2000** | | | | | | | | | | | | |
| Any diagnosis | 12.34 [0.11] | 14.50 [0.10] | 13.19 [0.11] | 15.86 [0.10] | -2,154 | <.001 | -0,851 | <.001 | -2,665 | <.001 | -1,362 | <0.001 |
| ADHD | 12.98 [0.11] | 14.58 [0.11] | 14.40 [0.11] | 15.61 [0.11] | -1,601 | <.001 | -1,419 | <.001 | -1,217 | <.001 | -1,035 | <0.001 |
| Anxiety Disorders | 13.08 [0.11] | 15.96 [0.11] | 13.98 [0.11] | 16.84 [0.11] | -2,885 | <.001 | -0,898 | <.001 | -2,864 | <.001 | -0,877 | <0.001 |
| Depressive Disorders | 13.15 [0.11] | 16.60 [0.11] | 14.21 [0.11] | 17.13 [0.11] | -3,450 | <.001 | -1,061 | <.001 | -2,918 | <.001 | -0,529 | <0.001 |
| OCD | 13.48 [0.11] | 15.82 [0.15] | 14.73 [0.11] | 16.64 [0.14] | -2,342 | <.001 | -1,248 | <.001 | -1,912 | <.001 | -0,818 | <0.001 |
| Bipolar Disorder | 13.54 [0.11] | 15.01 [0.37] | 14.80 [0.11] | 16.93 [0.27] | -1,471 | <.001 | -1,256 | <.001 | -2,129 | <.001 | -1,915 | <0.001 |
| Psychotic Disorders | 13.55 [0.11] | 17.10 [0.35] | 14.81 [0.11] | 16.72 [0.35] | -3,551 | <.001 | -1,262 | <.001 | -1,910 | <.001 | 0,380 | 0.422 |
| Anorexia Nervosa | 13.52 [0.11] | 17.00 [0.37] | 14.75 [0.11] | 17.38 [0.15] | -3,477 | <.001 | -1,231 | <.001 | -2,625 | <.001 | -0,380 | 0.306 |
| OED | 13.53 [0.11] | 15.28 [0.33] | 14.74 [0.11] | 17.25 [0.15] | -1,753 | <.001 | -1,214 | <.001 | -2,508 | <.001 | -1,970 | <0.001 |
| Sleep Disorders | 13.49 [0.11] | 14.75 [0.13] | 14.71 [0.11] | 16.01 [0.13] | -1,268 | <.001 | -1,225 | <.001 | -1,296 | <.001 | -1,253 | <0.001 |
| Self-harm | 13.49 [0.11] | 14.90 [0.19] | 14.71 [0.11] | 16.82 [0.14] | -1,408 | <.001 | -1,213 | <.001 | -2,116 | <.001 | -1,92 | <0.001 |
| *Note.* Males are the reference. Abbreviations: ADHD – attention deficit hyperactivity disorder, OCD – obsessive-compulsive disorder, OED – other eating disorders. Data for borderline personality disorder could not be included in this analysis due to sample size. | | | | | | | | | | | | |

# Table S13. Birth-year adjusted mean ages among individuals with ID.

|  | **Mean ages [standard errors] of diagnosis adjusted for birth year** | | | | **Group comparisons** | | | | | | | |
| --- | --- | --- | --- | --- | --- | --- | --- | --- | --- | --- | --- | --- |
| **Diagnosis** | Males without a preceding diagnosis (M) | Males with a preceding diagnosis (M+) | Females without a preceding diagnosis (F) | Females with a preceding diagnosis (F+) | MM+ | P_MM+_ | MF | P_MF_ | FF+ | P_FF+_ | M+F+ | P_M+F+_ |
| Any diagnosis | 8.42 [0.11] | 12.82 [0.13] | 9.12 [0.14] | 14.16 [0.16] | -4,398 | <0.001 | -0,697 | <.001 | -5,044 | <.001 | -1,343 | <0.001 |
| ADHD | 8.80 [0.12] | 12.80 [0.14] | 9.87 [0.14] | 13.86 [0.18] | -4,002 | <0.001 | -1,070 | <.001 | -3,993 | <.001 | -1,061 | <0.001 |
| Anxiety Disorders | 9.69 [0.11] | 15.87 [0.28] | 10.35 [0.13] | 16.77 [0.24] | -6,185 | <0.001 | -0,661 | <.001 | -6,418 | <.001 | -0,894 | 0.011 |
| Depressive Disorders | 9.83 [0.12] | 16.48 [0.33] | 10.66 [0.13] | 16.77 [0.28] | -6,646 | <0.001 | -0,832 | <.001 | -6,113 | <.001 | -0,298 | 0.459 |
| OCD | 9.87 [0.12] | 15.13 [0.47] | 10.88 [0.14] | 16.17 [0.63] | -5,258 | <0.001 | -1,005 | <.001 | -5,293 | <.001 | -1,040 | 0.183 |
| Bipolar Disorder | 9.95 [0.12] | 14.13 [0.57] | 10.90 [0.14] | 17.41 [0.58] | -4,181 | <0.001 | -0,954 | <.001 | -6,512 | <.001 | -3,285 | <0.001 |
| Psychotic Disorders | 9.92 [0.12] | 17.39 [0.52] | 10.93 [0.14] | 16.04 [0.54] | -7,473 | <0.001 | -1,007 | <.001 | -5,116 | <.001 | 1,350 | 0.065 |
| Anorexia Nervosa | 9.96 [0.12] | 11.93 [3.85] | 10.94 [0.14] | 17.13 [0.72] | -1,970 | 0.609 | -0,980 | <.001 | -6,196 | <.001 | -5,207 | 0.184 |
| OED | 9.94 [0.12] | 12.45 [1.03] | 10.88 [0.14] | 16.33 [0.57] | -2,518 | 0.014 | -0,947 | <.001 | -5,450 | <.001 | -3,879 | <0.001 |
| Sleep Disorders | 9.85 [0.12] | 13.26 [0.30] | 10.74 [0.14] | 15.54 [0.37] | -3,418 | <0.001 | -0,893 | <.001 | -4,800 | <.001 | -2,275 | <0.001 |
| Self-harm | 9.92 [0.12] | 14.23 [0.47] | 10.80 [0.14] | 16.27 [0.41] | -4,307 | <0.001 | -0,878 | <.001 | -5,475 | <.001 | -2,046 | <0.001 |
| BPD | 9.97 [0.12] | 18.67 [2.07] | 10.93 [0.14] | 19.06 [0.57] | -8,699 | <0.001 | -0,963 | <.001 | -8,122 | <.001 | -0,386 | 0.857 |
| *Note.* This table compares the birth year adjusted (reference year: 2000) mean age at autism diagnoses between autistic males with and without a preceding diagnosis (MM+), autistic males and females (MF), autistic females with and without a preceding diagnosis (FF+), and autistic males and females with a preceding diagnosis (M+F+). Abbreviations: ADHD – attention deficit hyperactivity disorder, OCD – obsessive-compulsive disorder, OED – other eating disorders, BPD – borderline personality disorder. | | | | | | | | | | | | |

# Table S14. Odds ratio of stability comparing males and females.

| N= 72,331 | **OR** | **lower CI** | **upper CI** | **p** |
| --- | --- | --- | --- | --- |
| **Crude model** | | | | |
| ADHD | 1.01 | 0.91 | 1.11 | 0.9 |
| Anxiety Disorders | 1.55 | 1.44 | 1.67 | <.001 |
| Depressive Disorders | 1.08 | 1.00 | 1.17 | 0.047 |
| Obsessive-Compulsive Disorder | 0.96 | 0.81 | 1.14 | 0.669 |
| Bipolar Disorders | 1.03 | 0.79 | 1.34 | 0.82 |
| Psychotic Disorders | 0.61 | 0.45 | 0.82 | 0.001 |
| Anorexia Nervosa | 1.96 | 1.13 | 3.41 | 0.017 |
| Other Eating Disorders | 1.54 | 1.07 | 2.19 | 0.019 |
| Sleep Disorders | 1.56 | 1.41 | 1.73 | <.001 |
| Self-harm | 2.58 | 2.10 | 3.16 | <.001 |
| Borderline Personality Disorder | 2.36 | 1.31 | 4.24 | 0.004 |
| **Adjusted for birth year** | | | | |
| ADHD | 1.05 | 0.95 | 1.15 | 0.381 |
| Anxiety Disorders | 1.54 | 1.43 | 1.66 | <.001 |
| Depressive Disorders | 1.09 | 1.00 | 1.18 | 0.043 |
| Obsessive-Compulsive Disorder | 0.96 | 0.81 | 1.13 | 0.607 |
| Bipolar Disorders | 1.02 | 0.78 | 1.33 | 0.867 |
| Psychotic Disorders | 0.60 | 0.44 | 0.81 | 0.001 |
| Anorexia Nervosa | 1.90 | 1.08 | 3.34 | 0.026 |
| Other Eating Disorders | 1.30 | 0.90 | 1.89 | 0.163 |
| Sleep Disorders | 1.45 | 1.30 | 1.62 | <.001 |
| Self-harm | 2.37 | 1.93 | 2.90 | <.001 |
| Borderline Personality Disorder | 2.42 | 1.35 | 4.37 | 0.003 |
| *Note.* Males are the reference group. Abbreviations: ADHD – attention deficit hyperactivity disorder. | | | | |

# Table S15. Sensitivity analyses for stability.

| **Sensitivity analysis** | **S2^a^** | | **S3^b^** | | **S4^c^** | | **S5^d^** | |
| --- | --- | --- | --- | --- | --- | --- | --- | --- |
| **N** | N= 37,291 | | N= 45,084 | | N= 58,152 | | N= 39,513 | |
|  | **OR [95% CI]** | **p** | **OR [95% CI]** | **p** | **OR [95% CI]** | **p** | **OR [95% CI]** | **p** |
| **Adjusted for birth year** | | |  |  |  |  |  |  |
| ADHD | 1.11 [0.95, 1.29] | 0.194 | 0.94 [0.82, 1.06] | 0.312 | 0.98 [0.86, 1.11] | 0.708 | 0.85 [0.72, 0.99] | 0.037 |
| Anxiety Disorders | 1.67 [1.46, 1.91] | <.001 | 1.50 [1.36, 1.66] | <.001 | 1.81 [1.62, 2.02] | <.001 | 1.65 [1.44, 1.88] | <.001 |
| Depressive Disorders | 1.25 [1.09, 1.43] | 0.001 | 1.12 [1.01, 1.25] | 0.033 | 1.27 [1.12, 1.44] | <.001 | 1.16 [0.98, 1.36] | 0.078 |
| OCD | 1.06 [0.81, 1.40] | 0.678 | 0.96 [0.77, 1.19] | 0.699 | 0.96 [0.77, 1.20] | 0.708 | 0.89 [0.67, 1.18] | 0.405 |
| Bipolar Disorders | 1.39 [0.87, 2.21] | 0.164 | 1.01 [0.69, 1.48] | 0.948 | 1.48 [0.85, 3.36] | 0.171 | 0.99 [0.45, 2.17] | 0.987 |
| Psychotic Disorders | 0.64 [0.40, 1.02] | 0.060 | 0.77 [0.53, 1.10] | 0.152 | 0.74 [0.39, 1.39] | 0.347 | 0.44 [0.14, 1.33] | 0.145 |
| Anorexia Nervosa | 1.28 [0.60, 2.72] | 0.524 | 1.55 [0.81, 2.96] | 0.181 | 1.69 [0.85, 3.36] | 0.133 | 3.19 [1.13, 9.02] | 0.029 |
| Other Eating Disorders | 1.70 [0.99, 2.93] | 0.055 | 1.45 [0.92, 2.27] | 0.106 | 1.32 [0.79, 2.18] | 0.286 | 0.62 [0.30, 1.29] | 0.199 |
| Sleep Disorders | 1.54 [1.30, 1.83] | <.001 | 1.38 [1.19, 1.61] | <.001 | 1.35 [1.14, 1.60] | <.001 | 1.22 [0.99, 1.50] | 0.067 |
| Self-harm | 2.63 [1.99, 3.49] | <.001 | 2.83 [2.04, 3.93] | <.001 | 3.89 [2.79, 5.41] | <.001 | 4.78 [2.73, 8.36] | <.001 |
| BPD | 3.44 [1.07, 11.10] | 0.039 | 3.29 [1.38, 7.86] | 0.007 | * | * | * |  |
| *Note.* Males are the reference. Abbreviations: CI = confidence interval, ADHD – attention deficit hyperactivity disorder, OCD – obsessive-compulsive disorder, BPD – borderline personality disorder. * Data for borderline personality disorder could not be included in this analysis due to sample size. ^a^ Stability analyses in individuals with complete follow-up for 5 years after autism diagnosis.  ^b^ Stability analyses in those individuals that receive the psychiatric diagnosis at least twice after autism diagnosis.  ^c^ Includes only those diagnosed with autism before age 18.  ^d^ Includes only individuals born after 2000. | | | | | | | | |

# Table S16. Stability of diagnoses by autism diagnosis age groups.

|  | **OR** | **lower CI** | **upper CI** | **p** |
| --- | --- | --- | --- | --- |
| **Autism diagnosis age 1-8** (N= 20 275) | | | | |
| Any Disorder | 0.89 | 0.8 | 0.99 | 0.033 |
| ADHD | 0.85 | 0.73 | 0.99 | 0.033 |
| Anxiety Disorders | 2.01 | 1.75 | 2.32 | <.001 |
| Depressive Disorders | 1.49 | 1.26 | 1.76 | <.001 |
| Obsessive-Compulsive Disorder | 0.89 | 0.67 | 1.18 | 0.422 |
| Bipolar Disorders | * | * | * | * |
| Psychotic Disorders | * | * | * | * |
| Anorexia Nervosa | * | * | * | * |
| Other Eating Disorders | 0.67 | 0.34 | 1.32 | 0.246 |
| Sleep Disorders | 1.33 | 1.06 | 1.67 | 0.013 |
| Self-harm | 5.24 | 3.28 | 8.37 | <.001 |
| Borderline Personality Disorder | * | * | * | * |
| **Autism diagnosis age 9-15** (N= 29 922) | | | | |
| Any Disorder | 0.89 | 0.8 | 0.99 | 0.033 |
| ADHD | 0.85 | 0.73 | 0.99 | 0.033 |
| Anxiety Disorders | 2.01 | 1.75 | 2.32 | <.001 |
| Depressive Disorders | 1.49 | 1.26 | 1.76 | <.001 |
| Obsessive-Compulsive Disorder | 0.89 | 0.67 | 1.18 | 0.422 |
| Bipolar Disorders | 1.16 | 0.55 | 2.45 | 0.701 |
| Psychotic Disorders | 0.95 | 0.34 | 2.64 | 0.924 |
| Anorexia Nervosa | 1.72 | 0.68 | 4.34 | 0.254 |
| Other Eating Disorders | 0.67 | 0.34 | 1.32 | 0.246 |
| Sleep Disorders | 1.33 | 1.06 | 1.67 | 0.013 |
| Self-harm | 5.24 | 3.28 | 8.37 | <.001 |
| Borderline Personality Disorder | * | * | * | * |
| **Autism diagnosis age 16-24** (N= 19 559) | | | | |
| Any Disorder | 1.53 | 1.39 | 1.68 | <.001 |
| ADHD | 1.54 | 1.33 | 1.78 | <.001 |
| Anxiety Disorders | 1.47 | 1.33 | 1.63 | <.001 |
| Depressive Disorders | 1.04 | 0.94 | 1.15 | 0.491 |
| Obsessive-Compulsive Disorder | 1.02 | 0.8 | 1.3 | 0.85 |
| Bipolar Disorders | 1.18 | 0.82 | 1.68 | 0.37 |
| Psychotic Disorders | 0.58 | 0.4 | 0.85 | 0.005 |
| Anorexia Nervosa | 2.91 | 1.19 | 7.08 | 0.019 |
| Other Eating Disorders | 1.8 | 1.09 | 2.99 | 0.023 |
| Sleep Disorders | 1.6 | 1.37 | 1.87 | <.001 |
| Self-harm | 2.06 | 1.6 | 2.66 | <.001 |
| Borderline Personality Disorder | 2.62 | 1.18 | 5.85 | 0.018 |
| **Autism diagnosis age 25 and older** (N= 2575) | | | | |
| Any Disorder | 1.23 | 0.98 | 1.54 | 0.070 |
| ADHD | 0.97 | 0.66 | 1.42 | 0.873 |
| Anxiety Disorders | 1.28 | 1.03 | 1.60 | 0.027 |
| Depressive Disorders | 0.86 | 0.69 | 1.08 | 0.191 |
| Obsessive-Compulsive Disorder | 1.26 | 0.69 | 2.31 | 0.445 |
| Bipolar Disorders | 0.81 | 0.46 | 1.43 | 0.474 |
| Psychotic Disorders | 0.54 | 0.28 | 1.05 | 0.071 |
| Anorexia Nervosa | 1.37 | 0.23 | 7.98 | 0.727 |
| Other Eating Disorders | 2.18 | 0.50 | 9.52 | 0.300 |
| Sleep Disorders | 1.45 | 1.11 | 1.90 | 0.006 |
| Self-harm | 1.23 | 0.66 | 2.32 | 0.512 |
| Borderline Personality Disorder | 2.21 | 0.84 | 5.79 | 0.108 |
| *Note.* Males are the reference category. Abbreviations: ADHD – attention deficit hyperactivity disorder. *Not calculated as there were less than 5 individuals in each group. | | | | |

# Table S17. Sensitivity analysis with different time intervals between psychiatric diagnoses prior and post-autism diagnosis.

|  | **OR** | **lower CI** | **upper CI** | **p** |
| --- | --- | --- | --- | --- |
| **Maximum 10 years (5 prior, 5 post)** | | | | |
| ADHD | 1.01 | 0.91 | 1.13 | 0.799 |
| Anxiety Disorders | 1.51 | 1.39 | 1.63 | <.001 |
| Depressive Disorders | 1.09 | 1.01 | 1.19 | 0.032 |
| Obsessive-Compulsive Disorder | 0.94 | 0.79 | 1.12 | 0.503 |
| Bipolar Disorders | 1.06 | 0.80 | 1.40 | 0.681 |
| Psychotic Disorders | 0.56 | 0.41 | 0.77 | <.001 |
| Anorexia Nervosa | 1.77 | 0.99 | 3.18 | 0.054 |
| Other Eating Disorders | 1.34 | 0.91 | 1.98 | 0.144 |
| Sleep Disorders | 1.38 | 1.23 | 1.55 | <.001 |
| Self-harm | 2.12 | 1.70 | 2.64 | <.001 |
| Borderline Personality Disorder | 2.35 | 1.29 | 4.29 | 0.005 |
| **Maximum 7 years (5 prior, 2 post)** | | | | |
| ADHD | 1.04 | 0.95 | 1.14 | 0.395 |
| Anxiety Disorders | 1.45 | 1.34 | 1.57 | <.001 |
| Depressive Disorders | 1.07 | 0.98 | 1.16 | 0.138 |
| Obsessive-Compulsive Disorder | 1.01 | 0.85 | 1.20 | 0.916 |
| Bipolar Disorders | 1.00 | 0.76 | 1.31 | 0.976 |
| Psychotic Disorders | 0.62 | 0.46 | 0.86 | 0.003 |
| Anorexia Nervosa | 1.93 | 1.06 | 3.53 | 0.032 |
| Other Eating Disorders | 1.39 | 0.93 | 2.09 | 0.110 |
| Sleep Disorders | 1.38 | 1.22 | 1.55 | <.001 |
| Self-harm | 2.22 | 1.73 | 2.83 | <.001 |
| Borderline Personality Disorder | 1.98 | 1.09 | 3.59 | 0.025 |
| **Maximum 7 years (2 prior, 5 post)** | | | | |
| ADHD | 0.96 | 0.85 | 1.08 | 0.484 |
| Anxiety Disorders | 1.50 | 1.38 | 1.63 | <.001 |
| Depressive Disorders | 1.09 | 0.99 | 1.19 | 0.073 |
| Obsessive-Compulsive Disorder | 1.00 | 0.82 | 1.21 | 0.960 |
| Bipolar Disorders | 0.93 | 0.68 | 1.27 | 0.663 |
| Psychotic Disorders | 0.63 | 0.45 | 0.89 | 0.009 |
| Anorexia Nervosa | 1.57 | 0.82 | 3.01 | 0.176 |
| Other Eating Disorders | 1.26 | 0.81 | 1.95 | 0.298 |
| Sleep Disorders | 1.31 | 1.14 | 1.49 | <.001 |
| Self-harm | 2.27 | 1.76 | 2.92 | <.001 |
| Borderline Personality Disorder | 2.60 | 1.36 | 4.97 | 0.004 |
| **Maximum 4 years (2 prior, 2 post)** | | | | |
| ADHD | 1.01 | 0.91 | 1.12 | 0.875 |
| Anxiety Disorders | 1.44 | 1.32 | 1.56 | <.001 |
| Depressive Disorders | 1.06 | 0.96 | 1.16 | 0.237 |
| Obsessive-Compulsive Disorder | 1.07 | 0.89 | 1.30 | 0.459 |
| Bipolar Disorders | 0.91 | 0.67 | 1.23 | 0.526 |
| Psychotic Disorders | 0.71 | 0.50 | 1.00 | 0.048 |
| Anorexia Nervosa | 1.74 | 0.90 | 3.37 | 0.100 |
| Other Eating Disorders | 1.34 | 0.86 | 2.10 | 0.200 |
| Sleep Disorders | 1.33 | 1.16 | 1.52 | <.001 |
| Self-harm | 2.40 | 1.82 | 3.16 | <.001 |
| Borderline Personality Disorder | 2.12 | 1.12 | 4.01 | 0.022 |
| *Note.* Males are the reference. ADHD = attention deficit hyperactivity disorder. | | | | |

# Table S18. Odds ratio of the stability among autistic individuals with intellectual disability.

| N= 10,331 | **OR** | **lower CI** | **upper CI** | **p** |
| --- | --- | --- | --- | --- |
| **Crude model** | | | | |
| ADHD | 0.93 | 0.72 | 1.21 | 0.605 |
| Anxiety Disorders | 2.18 | 1.51 | 3.15 | <.001 |
| Depressive Disorders | 1.82 | 1.14 | 2.89 | 0.011 |
| Obsessive-Compulsive Disorder | 0.91 | 0.42 | 1.98 | 0.806 |
| Bipolar Disorders | 1.10 | 0.38 | 3.23 | 0.859 |
| Psychotic Disorders | 0.74 | 0.34 | 1.64 | 0.459 |
| Other Eating Disorders | 4.58 | 0.91 | 23.05 | 0.065 |
| Sleep Disorders | 1.94 | 1.32 | 2.86 | 0.001 |
| Self-harm | 3.82 | 1.90 | 7.68 | <.001 |
| **Adjusted for birth year** | | | | |
| ADHD | 0.95 | 0.73 | 1.25 | 0.735 |
| Anxiety Disorders | 2.06 | 1.42 | 2.99 | <.001 |
| Depressive Disorders | 1.76 | 1.10 | 2.82 | 0.018 |
| Obsessive-Compulsive Disorder | 0.98 | 0.42 | 2.31 | 0.969 |
| Bipolar Disorders | 1.06 | 0.33 | 3.39 | 0.916 |
| Psychotic Disorders | 0.79 | 0.33 | 1.89 | 0.598 |
| Other Eating Disorders | 5.43 | 0.87 | 34.05 | 0.071 |
| Sleep Disorders | 1.68 | 1.13 | 2.50 | 0.010 |
| Self-harm | 3.22 | 1.54 | 6.76 | 0.002 |
| *Note.* Males are the reference. ADHD = attention deficit hyperactivity disorder. Data for borderline personality disorder could not be included in this analysis due to sample size. | | | | |

# Figure S1. Cohort selection process.


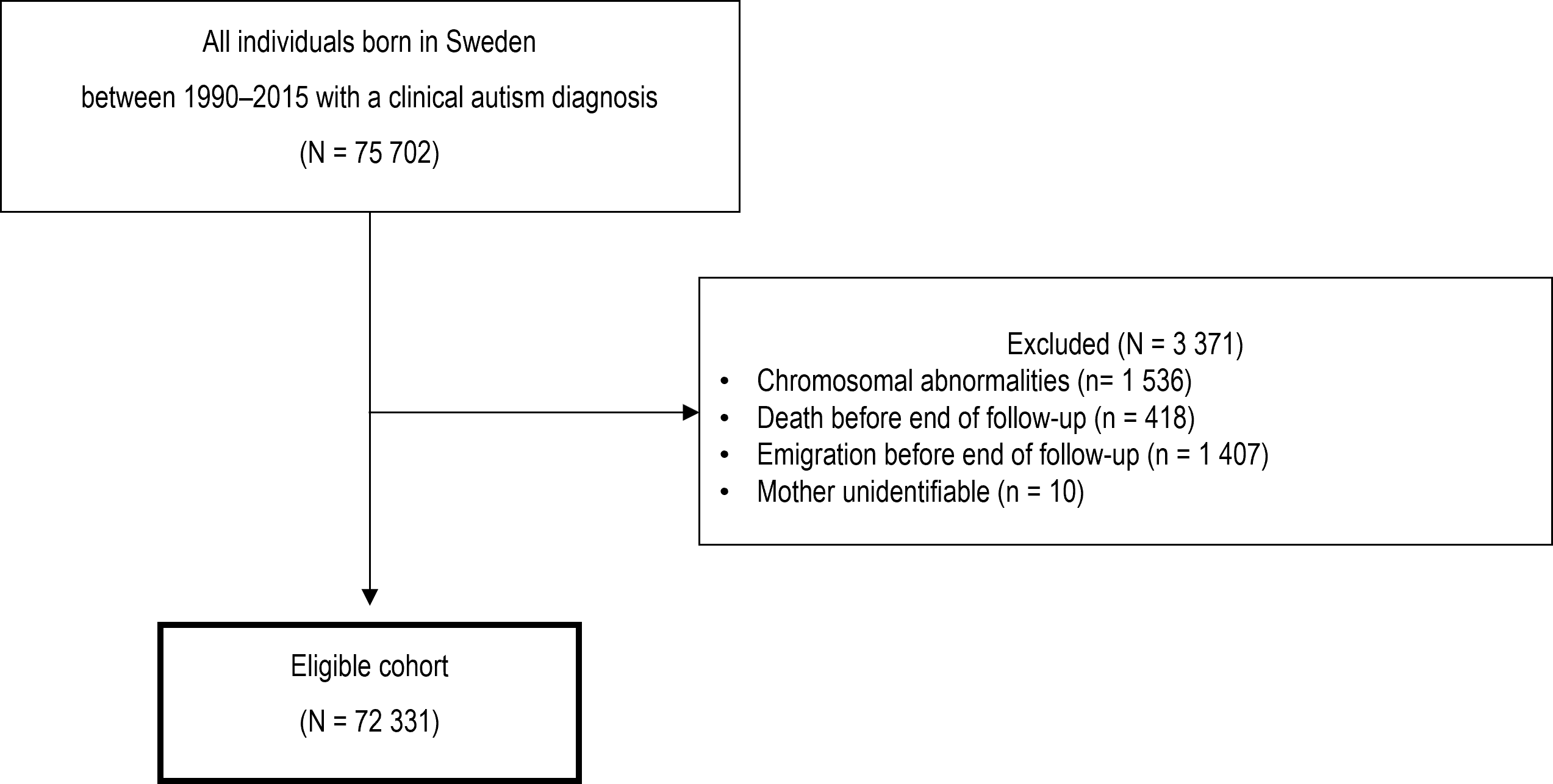


#
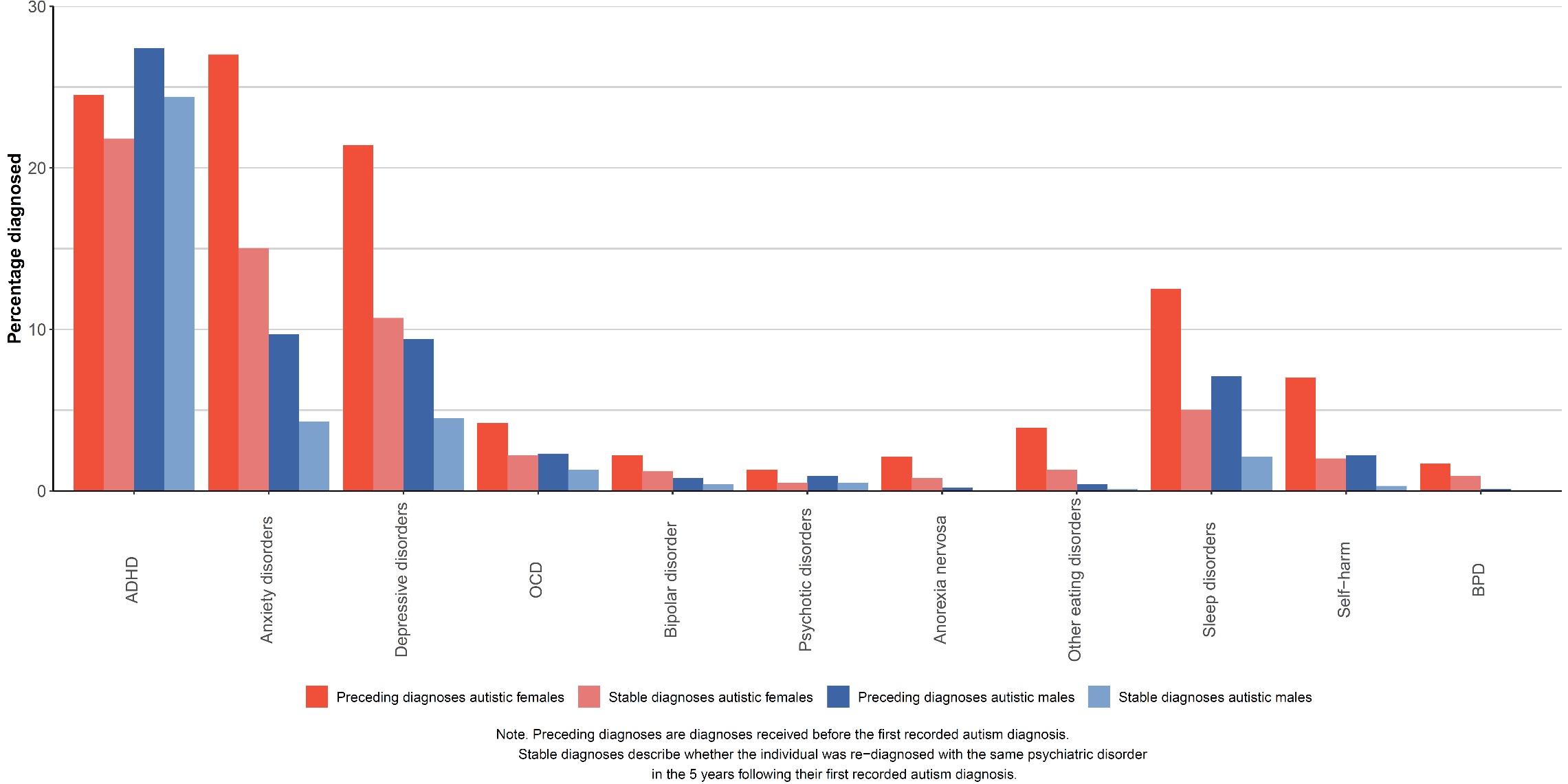
Figure S2. Proportion of autistic individuals with preceding and stable psychiatric diagnoses.

# Figure S3. Probability of preceding diagnoses in autistic females and males diagnosed with autism from 2010 to 2020.


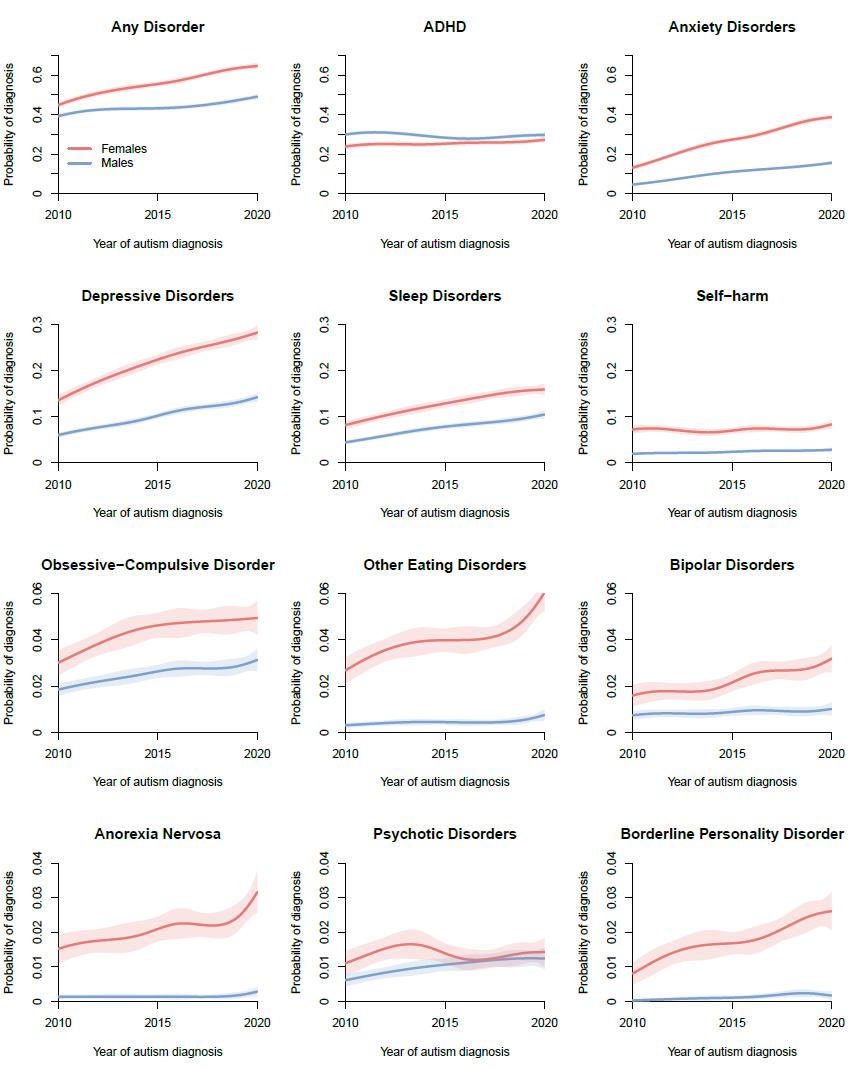


*Note.* Please note the differing y-axis by row. Abbreviations: ADHD – attention deficit hyperactivity disorder.

# Figure S4. OR comparing preceding diagnoses between females and males diagnosed with autism between 2010-2020 adjusted for age at autism diagnosis.


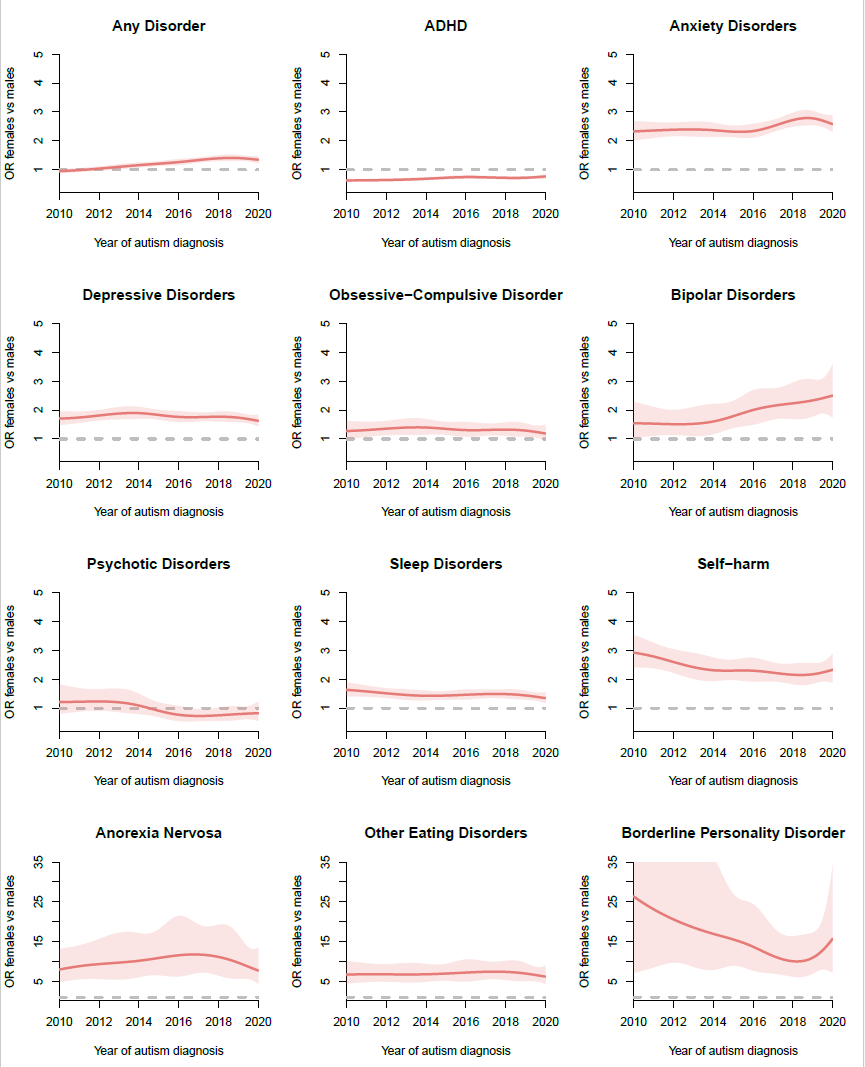


*Note.* Males are the reference group. Please note the difference in y-axis for anorexia nervosa, other eating disorders and borderline personality disorder. Abbreviations: ADHD – attention deficit hyperactivity disorder.
